# Butyrate Produced by Commensal Bacteria Down-Regulates *Indolamine 2,3-Dioxygenase 1* (*IDO-1*) Expression *via* a Dual Mechanism in Human Intestinal Epithelial Cells

**DOI:** 10.3389/fimmu.2018.02838

**Published:** 2018-12-11

**Authors:** Camille Martin-Gallausiaux, Pierre Larraufie, Anne Jarry, Fabienne Béguet-Crespel, Ludovica Marinelli, Florence Ledue, Frank Reimann, Hervé M. Blottière, Nicolas Lapaque

**Affiliations:** ^1^Micalis Institute, INRA, AgroParisTech, Université Paris-Saclay, Jouy-en-Josas, France; ^2^IFD, Sorbonne Universités, UPMC Univ Paris 06, Paris, France; ^3^MRC Metabolic Diseases Unit and Metabolic Research Laboratories, Wellcome Trust-MRC Institute of Metabolic Science, University of Cambridge, Cambridge, United Kingdom; ^4^CRCINA, INSERM, Université d'Angers, Université de Nantes, Nantes, France; ^5^US 1367 MetaGenoPolis, INRA, Université Paris-Saclay, Jouy-en-Josas, France

**Keywords:** gut microbiota, IDO-1, intestinal epithelial cells, butyrate, immune gene regulation

## Abstract

Commensal bacteria are crucial for the development and maintenance of a healthy immune system therefore contributing to the global well-being of their host. A wide variety of metabolites produced by commensal bacteria are influencing host health but the characterization of the multiple molecular mechanisms involved in host-microbiota interactions is still only partially unraveled. The intestinal epithelial cells (IECs) take a central part in the host-microbiota dialogue by inducing the first microbial-derived immune signals. Amongst the numerous effector molecules modulating the immune responses produced by IECs, indoleamine 2,3-dioxygenase-1 (IDO-1) is essential for gut homeostasis. *IDO-1* expression is dependent on the microbiota and despites its central role, how the commensal bacteria impacts its expression is still unclear. Therefore, we investigated the impact of individual cultivable commensal bacteria on *IDO-1* transcriptional expression and found that the short chain fatty acid (SCFA) butyrate was the main metabolite controlling *IDO-1* expression in human primary IECs and IEC cell-lines. This butyrate-driven effect was independent of the G-protein coupled receptors GPR41, GPR43, and GPR109a and of the transcription factors SP1, AP1, and PPARγ for which binding sites were reported in the *IDO-1* promoter. We demonstrated for the first time that butyrate represses *IDO-1* expression by two distinct mechanisms. Firstly, butyrate decreases STAT1 expression leading to the inhibition of the IFNγ-dependent and phosphoSTAT1-driven transcription of *IDO-1*. In addition, we described a second mechanism by which butyrate impairs *IDO-1* transcription in a STAT1-independent manner that could be attributed to its histone deacetylase (HDAC) inhibitor property. In conclusion, our results showed that *IDO-1* expression is down-regulated by butyrate *via* a dual mechanism: the reduction of STAT1 level and the HDAC inhibitor property of SCFAs.

## Introduction

The gut microbiome is a microbial ecosystem that exerts diverse functions often associated with beneficial physiological effects for its host. Among these essential functions, the intestinal microbiome provides an extended repertoire of molecules that influences the host health notably *via* the development and the maturation of its immune system (1, 2). The molecular bases of the host-microbiota interactions are only just beginning to be unraveled and are mediated by a wide variety of metabolites produced by commensal bacteria (2, 3). Many bacteria-derived metabolites originate from dietary sources. Among them, an important role has been attributed to the metabolites derived from the bacterial fermentation of dietary fibers, namely the short chain fatty acids (SCFAs) linking host nutrition to immune development and functions (2, 3). Human cells respond to SCFAs through a signaling activation cascade involving specific G-protein coupled receptors (GPR41, GPR43, and GPR109a) and through an epigenetic regulation of gene expression by the inhibition of lysine or histone deacetylases (HDACs) (4–6).

Numerous studies suggest that the close intimacy between the mucosal microbial populations and the host intestinal cells is central for the fine regulation of the host physiology. Indeed, intestinal epithelial cells (IEC) provide a crucial physical barrier against harmful pathogens and are also key players in the initiation and maintenance of mucosal immune responses (7). Accordingly, indigenous members of the microbiota have dramatic and specific impacts on the host immune system through their intimate interactions with the host epithelium (5, 8–11).

Indoleamine 2,3-dioxygenase-1 (IDO-1) is an enzyme that catalyzes the oxidation of the indole moiety of the essential amino acid tryptophan leading to production of N-formyl-kynurenine and its derivatives. In the last decades, a growing number of studies showed the importance of IDO-1 in various pathologies, including, autoimmune diseases, allergy, and cancer (12, 13). Despites the fact that IDO-1 expression was largely thought to be protective, several recent studies suggest a detrimental role of IDO-1 expression in obesity, atherosclerosis, vascular inflammation, and aneurysm (14–16). These results suggest that IDO-1 plays a far more complex role in health and fine-tuning of its expression and activity might occur in healthy individuals. Mechanisms inducing *IDO-1* expression during inflammation have already been described and include IFNγ and type-I IFN. However, natural factors inhibiting IDO-1 expression have not been reported yet.

The gut, along with the skin, is a major site of IDO-1 activity at steady state. IDO-1 expression in human healthy IECs is poorly described but has been reported in several studies to be increased in IBD (17–20). In the murine gut, its expression is dependent on the microbiota (10, 21). These observations prompted us to investigate the impact of individual cultivable commensal bacteria on *IDO-1* transcriptional expression. In the current study, we screened over 401 bacterial supernatants on an *IDO-1* reporter system and found that butyrate was the main inhibitor of *IDO-1* expression in human primary IECs and cell-lines. The *IDO-1* down-regulation was independent of GPR41, GPR43, and GPR109a, three known G-protein coupled receptors for SCFAs and of SP1, AP-1, and PPARγ, three transcription factors targeted by butyrate and for which binding sites were reported in the *IDO-1* promoter. Our results showed that butyrate regulated *IDO-1* expression *via* a dual mechanism. First, butyrate decreased STAT1 expression leading to the inhibition of the IFNγ-dependent phosphorylation of STAT1 and consequently the STAT1-driven transcriptional activity of *IDO-1*. In addition, we described a second mechanism by which butyrate impaired *IDO-1* transcription in a STAT1 independent manner that could be attributed to the HDAC inhibitory property of SCFAs.

## Materials and Methods

### IDO-1 Expression in Human Normal Colon at the Protein and mRNA Levels

Macroscopically and microscopically unaffected human normal colon was obtained from 10 patients undergoing surgery for colon cancer, at least at 10 cm downstream the tumor [7 men, 3 women; mean age 62 years; left (7) or right colon (3)]. The tissue fragments were processed accordingly to the French guidelines for research on human tissues, including patients' consent. IDO-1 immunostaining was performed using a monoclonal antibody (clone 4D2, Serotec) and a standard streptavidin-biotin-peroxidase technique after antigen retrieval in citrate buffer pH6. Diaminobenzidine was used as a chromogen and nuclei were counterstained with hematoxylin. *IDO-1* mRNA levels were assessed on preparations of isolated IECs after EDTA treatment and on whole mucosa microdissected from the normal colon as previously described (22). Samples were prepared by beads-beating mechanical lysis using Fastprep (MP Biomedicals) and centrifuged at 8,000 g for 10 min at 4°C prior RNA extraction and RT-PCR analysis.

### Cell Culture of Human Intestinal Cell Lines and Primary Colonocytes

The human epithelial cell lines HT-29 and Caco-2 were obtained from the American Type Culture Collection (ATCC, Rockville, MD) and grown as described (23). Four human primary colonic cell culture from three different donors were performed as described (24). Briefly, PBS-washed colonic tissues were digested with 0.5 mg/ml of collagenase type XI. The crypts were plated onto Matrigel coated plates and cultured for 24 h in DMEM 24 mM glucose supplemented with 10% FCS, 2 mM L-Glutamine, 50 U/mL penicillin, 50 U/mL streptomycin, and Y-27632 (Tocris). The day after plating, media was rinsed with fresh media and replaced with culture media with or without butyrate 2 mM. Human tissues were obtained from the Human Research Tissue Bank at the Addenbrooke's Hospital, Cambridge under the license 09/H0308/24.

### Luciferase Reporter and Cell Viability Assays

A 1.6-Kb section of the human *IDO-1* promoter was cloned using KpnI and NheI restrictions sites (Primers used were Fw: AAAGGTACCGGGTAGGATAGATTTAGTGAG; Rv: AAAAAGCTAGCCATTCTTGTAGTCTGCTCC) into the pGL4.14 (Promega) luciferase plasmid and used to establish the stable HT-29 *IDO-1* reporter cell-line after antibiotic selection (hygromycin, 600 μg/mL, InvivoGen) and validated with IFNγ (100 U/mL, Peprotech) and IL1β (10 ng/mL, Peprotech). For each experiment, HT-29-*IDO-1* reporter cells were seeded at 3 × 10^4^ cells per well in 96-well plates 24 h prior to incubation with bacterial supernatants or reagents. The cells were stimulated for 24 h with 10 μL of bacterial supernatants in a total culture volume of 100 μL per well (i.e., 10% vol/vol) prior to the luciferase assay. The luciferase activity was quantified as relative luminescence units using a microplate reader (Tecan) and the Neolite Luminescence Reporter Assay (Perkin-Elmer) according to the manufacturers' instructions. The *IDO-1* activity was normalized to the controls, i.e., the un-stimulated cells or cells in presence of non-inoculated bacteria culture medium. Experiments were performed in triplicates for at least three biological independent assays. Cell viability was monitored by MTS measurement using the CellTiter 96 Aqueous One solution (Promega) according to the manufacturer's recommendations.

### Culture of Commensal Bacteria, Preparation of Supernatants, and SCFAs Concentration Assessment

One hundred and thirty-five human intestinal commensal bacterial strains which include 111 different species from the in-house INRA-Micalis collection or from DSMZ were grown. Bacterial cultures and supernatants were performed as described (23). Screened species and strains, corresponding growth media, optical densities (OD), SCFAs concentrations are listed in Supplementary Table 1. Concentrations of SCFAs produced by cultured bacteria were measured by HPLC and gas chromatography as described (25).

### Reagents and Cytokines

All agonists, drugs and inhibitors were dissolved in glycerol, DMSO or water. Sodium salt of SCFAs were from Sigma and used in a range of concentrations from 0.5 to 8 mM. GPRs agonists: GPR41: 4-CMTB (1 μM Tocris) and Tiglic acid (1–10 mM Sigma); GPR43: AR420626 (1 μM Cayman) and 1-MCPC (1mM Sigma); GPR109a: Niacine (1–10 mM, Sigma) and MK1903 (1 μM Tocris). GPRs sub-unit inhibitors used were: Pertussis toxin (Ptx 0.2 μg/ml) and U73122 (10 μM) from Sigma. HDAC inhibitors: Trichostatin A (TSA 1 μM Sigma), SAHA (5 μM Sigma) and valproic acid (VPA 5 mM Sigma). SP1 inhibitor Mithramycin A (0.1 μM Sigma). PPARγ activators: Pioglitazone (5 μM), Roziglitazone (10 μM) and PPARγ inhibitor G9662 (100 μM), from Cayman. NF-kB inhibitor BAY 11-7082 (40 μM). AP-1 inhibitor SR-11302 (10 μM Tocris). STAT3/Jak2 inhibitor Cucurbitacin I (1 μM) from Tocris. IFNγ (100 U/ml) and TNFα (10 ng/ml) were from Peprotech. Final concentration of DMSO had no detectable effect on cells viability or responses.

### Plasmids and Transfection

Human GPR43 and GPR109a were cloned after EcoRI and XhoI digestion in pCMV-eGFP-N1 vector. Oligonucleotides used for amplification of GPR43 were *aaaactcgagatgctgccggactggaa* and *aaaagaattcctactctgtagtgaagtccga*. Oligonucleotides used for amplification of GPR109a were *aaaactcgagatgaatcggcaccatctgcaggat* and *aaaagaattcttaaggagaggttgggcccaga*. HT-29 cells were seeded at 3.10^4^ density per well in 96-well plates and transiently transfected with Lipofectamine 2000 (Thermofischer). 24 h after transfection, incubation with reagents was done for an additional 24 h prior luciferase activity measurement.

### siRNA Assays

HT29 cells were seeded at 4.10^5^ cells per well in a 6 wells plates on day 1 and siRNA were transfected with DharmaFect I at final concentrations of 1 and 25 nM on day 2 and 3, following the manufacturer's instructions (Dharmacom). Incubation with drugs was done on day 6 and *IDO-1* activity was assessed on day 7. siRNA SMARTpool ON-TARGETplus STAT1 siRNA (L-003543-00-0005) and ON-TARGETplus Non-targeting Pool (D-001810-10-05) were from Dharmacon.

### Real-Time PCR

Real-Time PCR were performed as described (23). qPCRs were carried out using a StepOnePlus Real-Time PCR System (ThermoFischer Scientific) with Taqman gene expression assay probes: *GAPDH* Hs02758991_g1, IDO-1 Hs00984148_m1, *GPR43* Hs00271142_s1, *GPR41* Hs02519193_g1, *GPR109a* Hs02341584_s1, *RBP1* Hs01011512_g1, *Actinbeta* Hs99999903_m1, *STAT1* s01013996_m1, *B2M* Hs99999907_m1. *GAPDH, Actin, RBP1* and B2M were used for internal normalization. Samples were tested in experimental duplicates and at least in biological triplicates. For primary cells treated with butyrate and control, cDNAs were pre-amplified (10 cycles) using the TaqMan PreAmp Matster Mix Kit following the manufacturer's recommendations.

### Western Blot Analysis

HT-29 cells were seeded at densities of 5 × 10^5^ cells per well in 24-well-plates for 24 h prior stimulation. Cells were washed twice and lysed in buffer (1% NP40, 150 mM NaCl, 50 mM Tris-HCL pH8, 5 mM EDTA, 1 × Complete Protease Inhibitor Cocktail (Roche), 1X × Phos STOP phosphatase Inhibitor Cocktail (Roche). Nucleus were eliminated by centrifugation for 10 min 4°C at 17,500 g. Protein extracts were run in SDS-PAGE gels and transferred onto PVDF membranes. Membranes were blocked overnight in TBS 0.1% tween 4% skim milk or BSA (Sigma). Primary antibodies were incubated overnight at 4°C (STAT1 1:1000 (D1K9Y), STAT1-phospho TYR 701 1:1,000 (58D6), STAT3 1:1,000 (124H6), Lamin A/C 1:2,000 (4C11) all from Cell signaling; Actin 1:2,000 (AC-40) from Sigma, GAPDH 1:2,000 from Santa Cruz). Secondary (Goat anti-Rabbit IgG HRP (P0448) and Goat anti-mousse HRP (P0447) from Dako) antibodies were successively added for 1h before detection with the Clarity Western ECL Substrate using the Chemidoc MP System (Bio-Rad). Quantifications were performed using the image Lab software (Bio-Rad). Proteins levels were internally normalized with GAPDH or Actin before comparison with experimental controls.

### Cytoplasmic and Nuclear Proteins Extraction

HT-29 cells were seeded at densities of 5 × 10^5^ cells/well in 24-well-plates for 24 h prior stimulation with butyrate. Cytoplasmic and nuclear protein extracts were prepared using the NE-PER Nuclear and Cytoplasmic Extraction Reagents according to the manufacturer's instructions (ThermoScientific). Lamin A/C and GAPDH were used as nuclear and cytoplasmic protein loading controls, respectively.

### Promoter Analysis

*in silico* analysis of the promoter sequence upstream of the transcription start of *IDO-1* was performed using Genomatix MatInspector software (core similarity = 1; matrix similarity > 0.8).

### Statistical Analysis

Data were analyzed using R and RStudio software. Function for PCA analysis: prcomp. Correlation matrix was done with Hmisc package. Graphics were produced with ggplot2 package and Prism GraphPad software. Statistical analysis was done with Student two-sided test or Wilcoxon rank test.

## Results

### IDO-1 Is Expressed in Epithelial Cells of the Human Normal Colonic Mucosa

**Figure 1:**
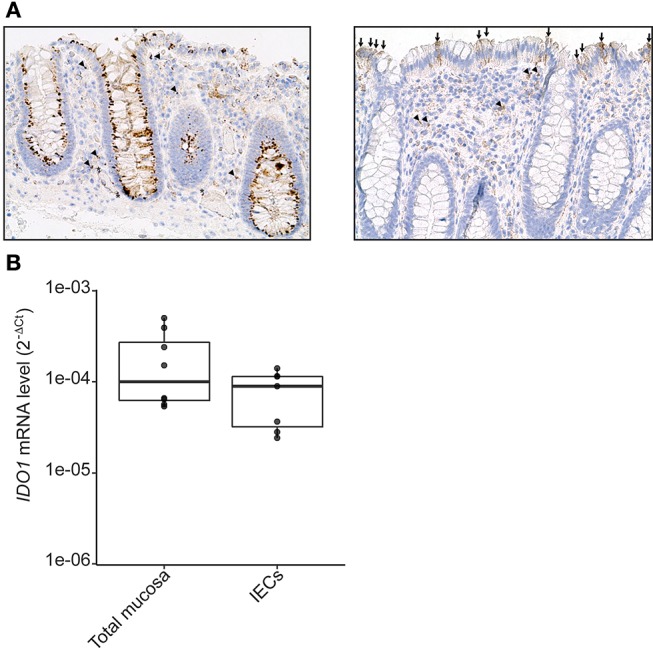
IDO-1 expression in human colonic epithelial cells. **(A)** Human normal colonic mucosa was stained for IDO-1. Representative immunohistochemical staining of IDO-1 showed that IDO-1 (brown) is expressed in epithelial cells [left: strong perinuclear and/or membrane staining of about 80% of the IECs; right panel: heterogeneous staining of few IECs (arrows)] and in few *lamina propria* mononuclear cells (arrowheads) and endothelial cells (asterisk) (original magnification × 200). **(B)**. *IDO-1* gene expression was determined by RT-PCR on RNA extracted from preparations of isolated human colonic epithelial cells (IECs) and of whole mucosa microdissected from normal colon. Results were normalized to β*-2 microglobulin* (*B2M*) and expressed as 2^*-ΔCt*^ relative value (median ± quartiles) of 4 patients (1–2 samples/patient).

IDO-1 expression is well-documented in dendritic cells (DC) and macrophages (26). However, its expression in IECs has been scarcely studied in human. We assessed IDO-1 expression both by immunohistochemistry on paraffin sections of normal human colonic mucosa (*n* = 10) and at the mRNA level. In 8 cases, IDO-1 was expressed by IECs with either strong homogeneous staining of more than 80% IECs all along the colonic crypts (perinuclear and/or membrane staining in enterocytes and goblet cells; Figure 1A, left panel) or heterogeneous staining of IECs (10–20% of IECs; Figure 1A, right panel). In 2 samples, IDO-1 was barely detectable in IECs. IDO-1 was also expressed in the *lamina propria*, in some mononuclear cells and endothelial cells (Figure 1A). *IDO-1* expression was then confirmed by RT-PCR on RNA extracted from preparations of isolated human IECs from normal colon. As shown in Figure 1B, isolated human IECs expressed an *IDO-1* level comparable with the expression level from the entire colonic mucosa suggesting that IECs were an important source of *IDO-1* mRNAs in the colon.

### Metabolites Derived From Commensal Bacteria Modulate *IDO-1* Expression

**Figure 2:**
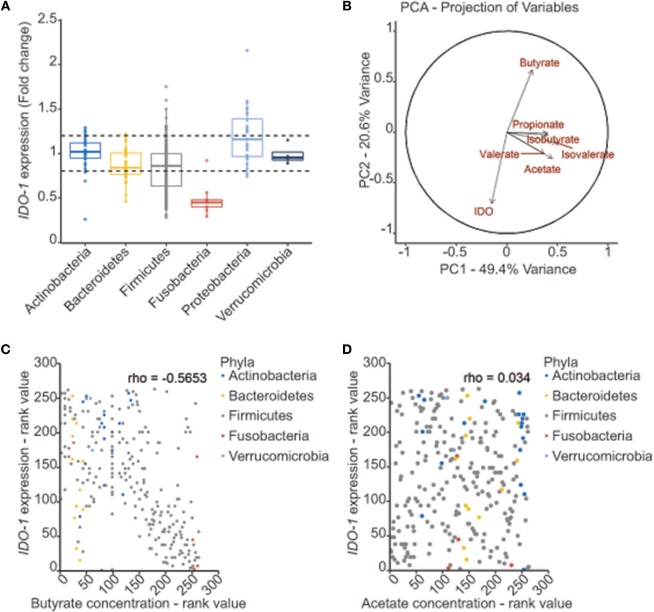
Correlation between bacterial metabolites production and *IDO-1* gene expression. **(A)** Effect of bacterial supernatants on *IDO-1* reporter system organized by phylum. Culture supernatants of a wide range of cultivable commensal bacteria were applied on the HT-29-*IDO-1* reporter system (10% vol/vol) for 24 h*. IDO-1* expression was measured by luciferase activity and expressed as fold increase toward its control: non inoculated growth medium used for each culture. *IDO-1* expression profiles upper and lower the dash lines were considered as significantly changed. **(B)** PCA analysis showing the correlation between the SCFAs concentrations produced by the commensal bacteria and *IDO-1* expression. **(C,D)** Representation of *IDO-1* expression correlated to butyrate **(C)** and acetate **(D)** concentration in in bacterial cultures classified by rank value. Actinobacteria in blue, Bacteroidetes in yellow, Firmicutes in gray, Fusobacteria in red and Verrucomicrobiea in light blue.

In the gut, *IDO-1* expression is dependent on the microbiota since colonization of mice with commensal bacteria induced high levels of *IDO-1* in IECs (10, 21). In an attempt to decipher which commensal bacteria influence *IDO-1* expression, we performed a screening with an *IDO-1* reporter system expressed in the human epithelial cell line HT-29. As recently reported in animal studies and in functional metagenomic studies, bioactive compounds produced by commensal bacteria are likely to be small-secreted molecules, we thus tested the bacterial supernatants of 135 members of the human microbiota that include 60% of species close to the human core microbiome on an *IDO-1* reporter system (Supplementary Table 1) (2, 3, 27, 28). In this set-up, only few bacterial supernatants were activating *IDO-1* expression in HT-29 cells, including some *Lactobacillaceae* (Supplementary Figure 1). Interestingly, a global and dramatic down-regulation of *IDO-1* was observed in HT-29 challenged with supernatants of Firmicutes and Fusobacteria, while Actinobacteria, Bacteroidetes, Proteobacteria, and Verrucomicrobia barely modulated *IDO-1* expression (Figure 2A and Supplementary Figure 1).

### Butyrate Down-Regulates *IDO-1* Expression in Epithelial Cells

Among the Firmicutes, the most active genera on *IDO-1* expression were *Clostridium, Lachnoclostridium, Ruminoclostridium*, and *Roseburia* (Supplementary Figure 1). All these genera in addition to the *Fusobacterium* genus share a common active role in the diet-derived fiber degradation leading to the production of short-chain fatty acids (SCFAs) by anaerobic fermentation (29). We thus hypothesized that the down-regulated pattern of *IDO-1* expression could be explained by the SCFA concentration in the bacterial supernatants. We therefore quantified the concentrations of acetate, propionate, butyrate, isobutyrate, valerate, and isovalerate by GC-MS or HPLC in some bacterial supernatants (Supplementary Table 1). Principal component (PCA) and correlation analyses on SCFAs concentrations and *IDO-1* activity showed a negative correlation between butyrate concentration and *IDO-1* expression (Figure 2B and Supplementary Figure 2A). Specific impact of butyrate on *IDO-1* was confirmed by a pairwise spearman correlation (Figure 2C). Analysis with acetate concentrations showed no correlation with *IDO-1* expression (Figure 2D).

**Figure 3:**
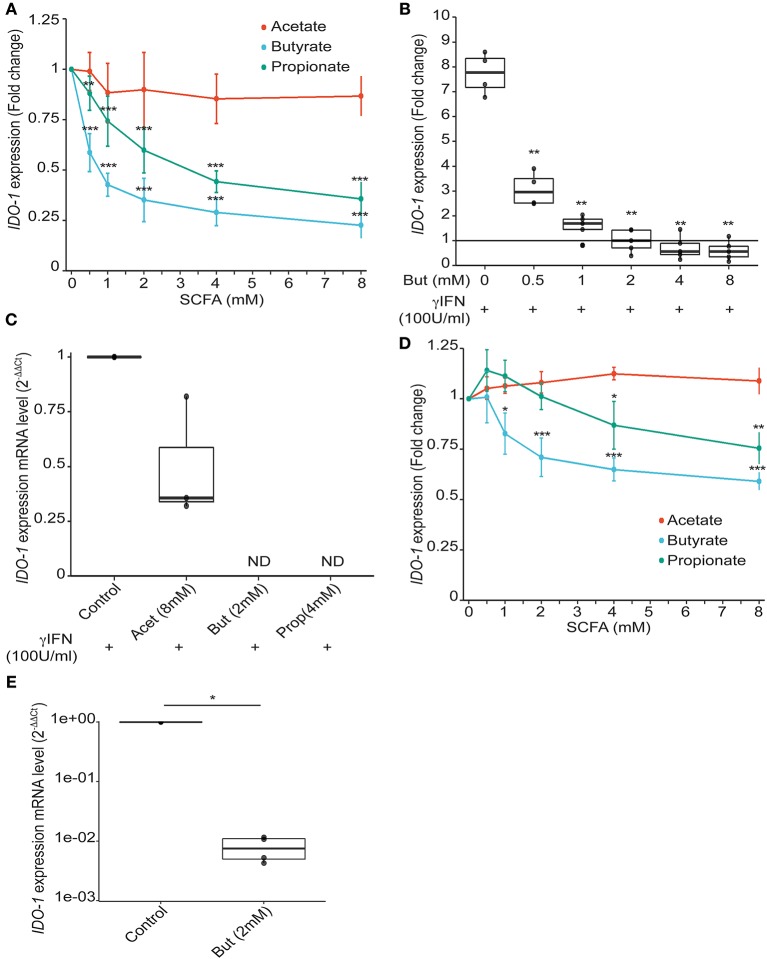
Impact of SCFAs on *IDO-1* expression. **(A)**, HT-29-*IDO-1* reporter cells were incubated with a range of concentration of acetate, butyrate and propionate (0.5; 1; 2; 4; 8 mM) for 24 h. *IDO-1* expression was measured by luciferase activity and expressed as the mean ± SD fold change toward un-stimulated cells (*N* > 3). **(B)**, HT-29-*IDO-1* reporter cells were incubated with IFNγ (100 U/ml) and a range of concentration of butyrate (0.5–8 mM). *IDO-1* expression was measured by luciferase activity and expressed as the median ± quartiles of fold change toward un-stimulated cells (*N* > 3). **(C)** *IDO-1* gene expression on HT-29 exposed for 6 h to IFNγ (100 U/ml) +/– butyrate (2 mM), propionate (4 mM), or acetate (8 mM) was determined by RT-PCR. Results were normalized to *GAPDH* and expressed as 2^*-ΔΔ Ct*^ relative to control mean value; ND: not detected (*N* = 3). **(D)** Caco2-*IDO-1* reporter cells were incubated with a range of concentration of acetate, propionate and butyrate (0.5; 1; 2; 4; 8 mM). *IDO-1* expression was measured by luciferase activity and expressed as the mean ± SD fold change toward un-stimulated cells (*N* > 3). **(E)** *IDO-1* expression level on human colonic epithelial cells treated for 24 h with butyrate compared to non-treated cells from the same patient was determined by RT-PCR. Results are normalized to *RPS17* and expressed as 2^*-ΔΔ Ct*^ relative to control, median ± quartiles (*N* = 4). *P*-value: *P < 0.05, **P < 0.005, ***P < 0.001.

We validated experimentally the observed correlations by testing the effect of a range of physiological intestinal concentration of SCFAs on *IDO-1* reporter system. Acetate which is the more abundant SCFA produced by gut bacteria had no impact on *IDO-1* expression. Butyrate and to a lesser extent propionate, isobutyrate, isovalerate, and valerate down-regulated *IDO-1* (Figure 3A and Supplementary Figure 2B). Indeed, as shown in Figure 3A, a significant *IDO-1* down-regulation was observed at a concentration as low as 0.5 mM for butyrate and propionate. These concentrations were consistent with the final SCFA concentrations in bacterial supernatants used in the screen thus supporting their involvement in *IDO-1* down-regulation (Supplementary Table 1). Butyrate and propionate are found in the human gut lumen at around 20 mM (30). Moreover, we showed that butyrate and propionate also inhibited Interferon γ (IFNγ)-induced *IDO-1* expression in a dose-dependent manner in our reporter system (Figure 3B and Supplementary Figure 2C). This result was confirmed at the mRNA level by RT-PCR in IFNγ-treated HT-29 cells with a total abolishment of *IDO-1* expression by butyrate and propionate while acetate had no significant impact (Figure 3C). In addition, the inhibitory impact of butyrate and propionate on *IDO-1* expression was observed in an *IDO-1* reporter system expressed in another IEC line, Caco-2 (Figure 3D and Supplementary Figure 2D). More importantly, we showed that this phenotype is not restricted to cell-lines as *IDO-1* mRNA level was also significantly down-regulated by butyrate in human primary colonocytes culture, compared to non-treated cells (Figure 3E).

### Butyrate Inhibits IFNγ-Induced *IDO-1* Expression by STAT1 Down-Regulation

**Figure 4:**
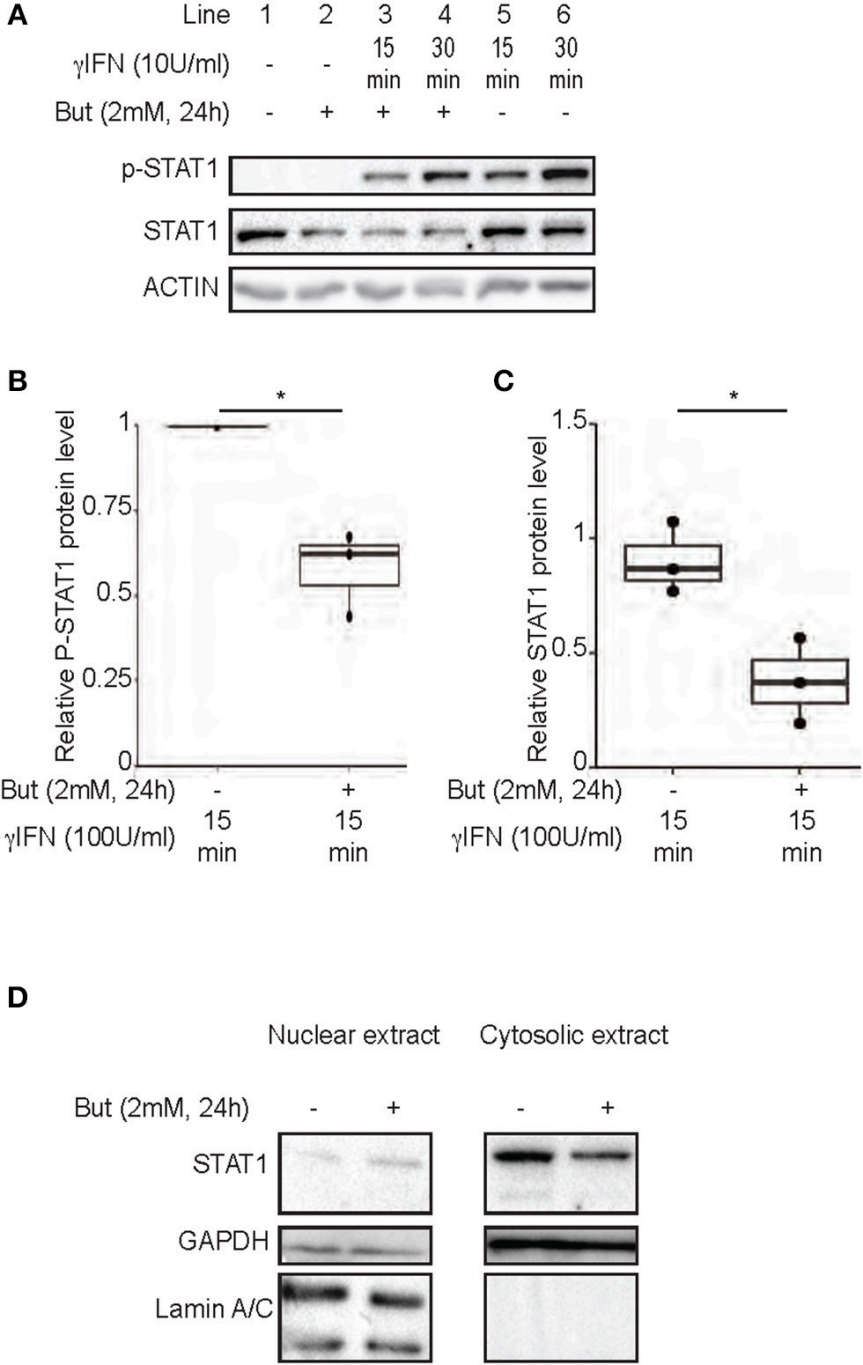
Inhibition of IFNγ-induced *IDO-1* expression by butyrate is correlated with a decrease of STAT1 protein level. **(A–C)** HT-29 cells were cultured 24 h with butyrate (But 2 mM) prior IFNγ (100 U/ml) stimulation for 15 (line 3 with butyrate and 5 without butyrate) or 30 min (line 4 with butyrate and 6 without butyrate). The protein level of p-STAT1 Tyr701, STAT1, and Actin were determined by western-blot on total protein extracted. Densitometric quantifications of total P-STAT1 and STAT1 proteins, from 3 independent experiments, were normalized to Actin and expressed as fold change compared to IFN stimulated cells **(B)** and unstimulated cell **(C)**, respectively, of 3 independent experiments. Data are represented as median ± quartiles. **(D)** HT-29 cells were incubated 24 h with medium or butyrate (But 2 mM) prior cytoplasmic and nuclear extractions. The protein levels of STAT1, Laminin A/C and GAPDH were assessed in each fraction by western-blot. *P*-value: *P < 0.05; NS, Non significant.

Several mechanisms of *IDO-1* induction have been reported. A classical cascade involves IFNγ-dependent phosphorylation of Signal transducer and activator of transcription 1 (STAT1) promoting *IDO-1* expression (31). Previous studies have demonstrated the inhibition of IFNγ-dependent phosphorylation of STAT1 by butyrate, in a nasopharyngeal carcinoma model (32, 33). We thus assayed by immunoblot analysis the impact of a 24 h-treatment of butyrate on the IFNγ-induced phosphorylation of STAT1 in HT-29 cells. In line with other studies, we observed less Tyr 701 phosphorylated form of STAT1 in cells pre-treated with butyrate (Figures 4A,B). Interestingly, in contrast to previous studies, we observed that this phenotype was directly correlated to a down-regulation of the protein level of STAT1 itself mediated by butyrate as both total STAT1 and phosphorylated STAT1 levels were similarly diminished (Figures 4A–C). The butyrate-driven STAT1 down-regulation was observed on both IFNγ stimulated and non-stimulated cells (Figures 4A,C and Supplementary Figure 3A). Interestingly, we did not monitor any inhibition of *STAT1* gene expression by RT-PCR at 6 and 24 h post incubation with butyrate (Supplementary Figure 3B) suggesting post-transcriptional modifications of STAT1. To further determine whether STAT1 was translocated in the nucleus by butyrate treatment, nuclear STAT1 protein level was assessed by immunoblotting in butyrate-treated and control HT-29 cells. As shown in Figure 4D, we did not detect accumulation of nuclear STAT1 in butyrate-treated cells. In summary, these findings demonstrated that butyrate strongly reduced STAT1 protein level which is a mechanism contributing to the inhibition of IFNγ-induced *IDO-1* in human IECs.

### Butyrate Inhibits *IDO-1* Expression Independently of STAT1 and STAT3

**Figure 5:**
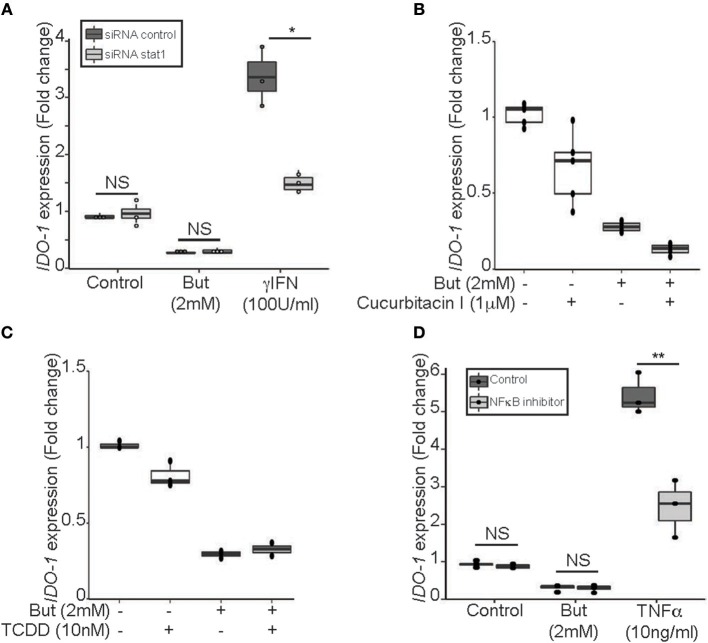
Butyrate inhibition of *IDO-1* promoter activity is STAT1 and STAT3 independent. **(A)** HT-29-*IDO-1* cells were transfected with STAT1 siRNA or control siRNA and incubated with butyrate (But 2 mM) or IFNγ (100 U/ml) for 24 h before measuring IDO-1 level. **(B)** HT-29-*IDO-1* cells were incubated for 2 h with the STAT3 phosphorylation inhibitor (Cucurbitacin I, 1 μM) prior to butyrate (But 2 mM) treatment for total incubation time of 24 h (*N* = 4). **(C)** HT-29-*IDO-1* cells were incubated with AHR ligand (TCDD 10 nM) +/– butyrate (But 2 mM) for 24 h. Data represented 2 independent experiments **(D)** HT-29-*IDO-1* cells were incubated for 1 h with the NκFB inhibitor, Bay117082 (Bay 40 μM) prior stimulation with butyrate (But 2 mM) or TNFα (10 ng/ml) for 24 h (*N* = 3). *IDO-1* expression was measured by luciferase activity and expressed as median ± quartiles of fold change toward unstimulated cells. Data represented at least three independent experiments. *P*-value: *P < 0.05, **P < 0.005, NS, Non significant.

To further decipher the mechanism of butyrate-driven *IDO-1* regulation observed in cells untreated with IFNγ, we studied STAT1 involvement in the *IDO-1* down-regulation observed in unstimulated IECs (Figure 3). The pivotal role of STAT1 was assayed using siRNA down-regulation (Supplementary Figure 4A). We observed no impact on butyrate-dependent inhibition of *IDO-1* in absence of STAT1 signaling. These results suggested that butyrate did not impact on basal STAT1-dependent signaling and that STAT1-independent mechanism may also be involved in *IDO-1* down-regulation (Figure 5A).

Two alternative pathways for *IDO-1* induction have been reported, involving STAT3 and aryl hydroxycarbon receptor (AHR) on one hand and an NFκB-dependent pathway on the other hand (34–36). We showed that blocking STAT3 phosphorylation (Cucurbitacin I) or activating AHR pathway (TCDD) did not induce *IDO-1* or prevent butyrate inhibition in our model supporting that the STAT3/AHR pathway was not involved in this process (Figures 5B,C). Moreover, immunoblotting assays on STAT3 level revealed, that in contrast to STAT1, STAT3 was not decreased following butyrate incubation for 24 h in HT-29 (Supplementary Figure 4B). In addition, we ruled out NFκB activation as NFκB inhibitor BAY 11-7082 did not impact on butyrate-driven *IDO-1* down-regulation, as positive control NFκB activation was induced by TNFα (Figure 5D). Altogether, these results suggested that butyrate down-regulated *IDO-1* independently of STAT1, STAT3, AHR, and NFκB.

### Butyrate-Mediated Impact on *IDO-1* Is Independent of the SCFAs Receptors GPR41, GPR43, and GPR109a

**Figure 6:**
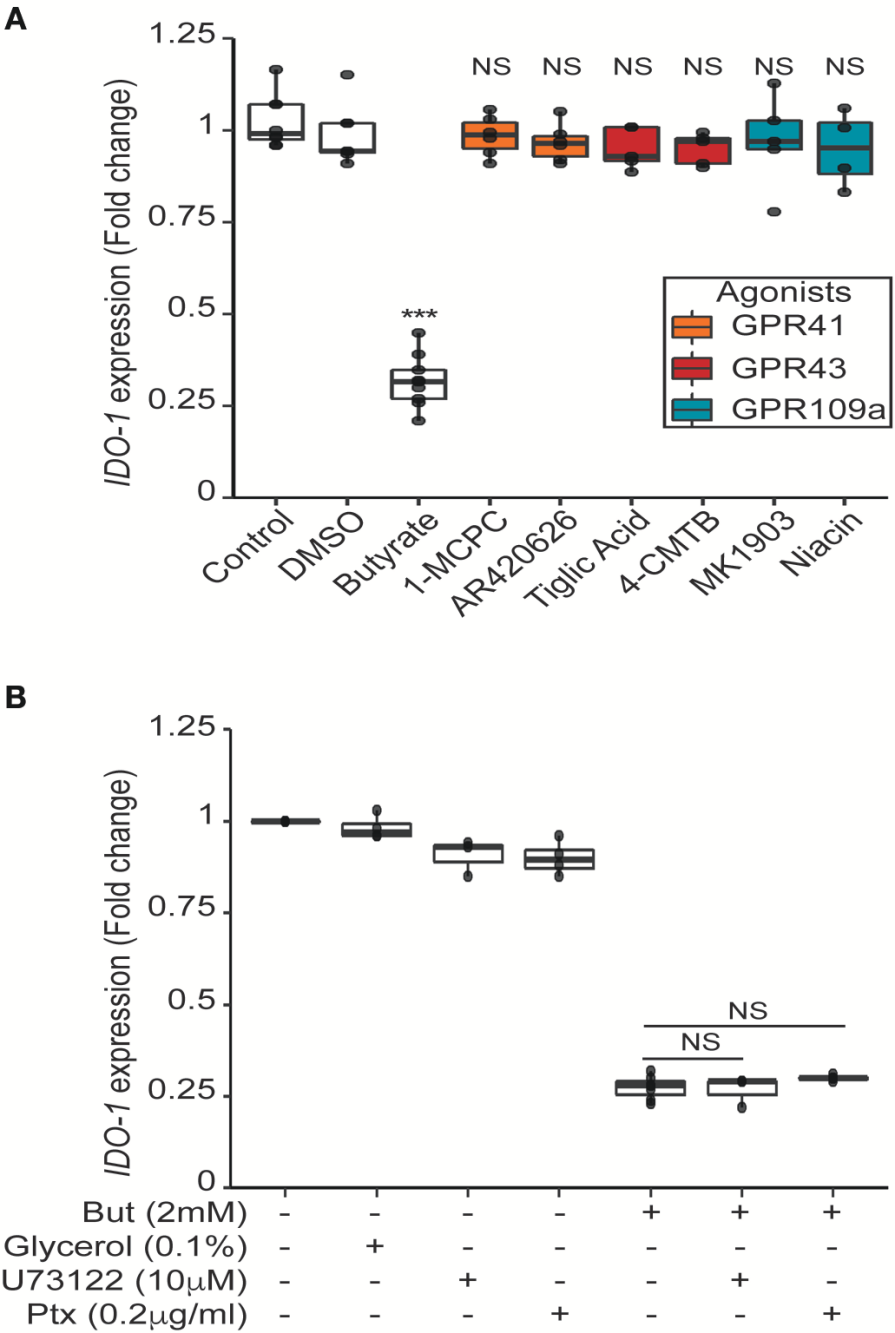
Butyrate mediated impact on *IDO-1* is independent of its receptors GPR41, GPR43 and GPR109a. **(A)** HT-29-*IDO-1* reporter cells were incubated for 24 h with selective GPR agonists: GPR41: AR420626 (1 μM) and 1-MCPC (1 mM); GPR43: 4-CMTB (1 μM) and Tiglic acid (1 mM); GPR109a: Niacin (1 mM) and MK1903 (1 μM) or with DMSO (vehicle), butyrate (But 2 mM) or Control (RPMI). **(B)** HT-29-*IDO-1* reporter cells were incubated for 24 h with 2 mM butyrate +/– GPRs sub-unit inhibitors: Pertussis toxin (Ptx, 0.2 μg/ml), U73122 (10 μM) or glycerol (vehicle). *IDO-1* expression was measured by luciferase activity and expressed as median ± quartiles of fold change toward un-stimulated cells. Data represented at least three independent experiments. *P*-value: ***P < 0.001, NS, Non significant.

Our data suggest that butyrate down-regulates *IDO-1* expression in a STAT1 and STAT3-independent manner and, thus, might involve an additional mechanism. SCFAs impact human cells through two main mechanisms: inhibition of histone and lysine deacetylases (K/HDAC) and activation of specific G-protein coupled receptors (GPR41, GPR109a: both Gα/i coupled receptors and GPR43: Gα/i and Gαq coupled receptor) (4, 5, 37). We confirmed that the three G-protein coupled receptors are expressed in HT-29 and Caco-2 cells (Supplementary Figures 5A,B). To test the potential role of these receptors, we first used selective agonists of GPR41 (1-MCPC and AR420626), GPR43 (Tiglic acid and 4-CMTB), and GPR109a (Niacin and MK1903). If the butyrate-driven down-regulation of *IDO-1* expression were mediated by the GPR-dependent signaling pathways, we should expect that activation of these receptors would inhibit *IDO-1* expression. Interestingly, none of these agonists, alone or in combination, impacted *IDO-1* expression (Figure 6A and Supplementary Figure 5C). To further confirm this observation, we used inhibitors of the Gαi and the Gαq pathways: the pertussis toxin (Ptx) and phospholipase Cβ inhibitor (U73122), respectively. As shown in Figure 6B, none of these inhibitors impacted on the butyrate-dependent *IDO-1* down-regulation. Moreover, over-expression of GPR43 and GPR109a in HT-29 did not impact the butyrate-dependent inhibition of *IDO-1* expression (Supplementary Figure 6). Altogether these results suggest that the SCFAs receptors GPR41, GPR43, and GPR109a were not involved in the observed butyrate-driven inhibition of *IDO-1* expression.

### Butyrate Down-Regulates *IDO-1* Expression *via* Its HDAC Inhibitory Property in an AP-1, PPARγ, and SP1-Independent Manner

**Figure 7:**
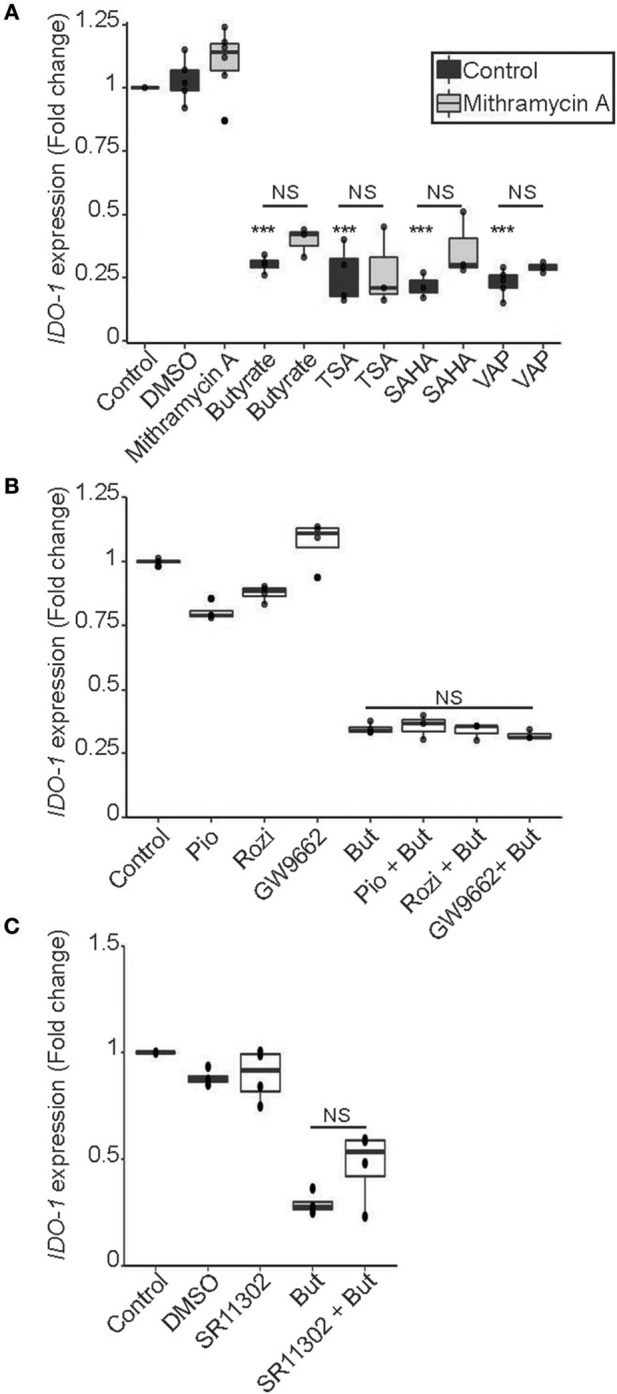
HDAC inhibitor mimicked the butyrate-dependent down-regulation of *IDO-1* expression in a SP1, PPARγ and AP-1 independent manner. **(A)** HT-29-*IDO-1* reporter cells were incubated for 24 h with butyrate (But 2 mM), SAHA (5 μM), Trichostatin A (TSA 1 μM) or Valproic acid (VAP 5 mM) ± SP1 inhibitor (Mitramycin A; MitA 0.1 μM). **(B)**, HT-29-*IDO-1* reporter cells were stimulated for 24 h with two PPARγ activators: Pioglitazone (Pio 5 μM); Rosiglitazone (Rosi, 10 μM) or the specific PPARγ inhibitor GW9662 (10 μM) ± butyrate (But 2 mM). **(C)** HT-29-*IDO-1* reporter cells were incubated for 24 h with butyrate (But 2mM) and/or the AP1 inhibitor, SR11302 (10 μM). *IDO-1* expression was measured by luciferase activity and expressed as median ± quartiles of fold change toward un-stimulated cells. Data represented at least three independent experiments. *P*-value: ***P < 0.001, NS, Non significant.

SCFAs, and butyrate in particular, are potent modulators of protein acetylation targeting histones and transcription factors. Indeed, SCFAs impact human cells through their ability to inhibit lysine and histone deacetylases (HDAC) and are thus considered as members of the HDAC inhibitor (HDACi) family (5, 38). As part of the aliphatic family of HDACi, butyrate targets HDAC class I (HDAC 1, 2, 3, 8) and IIa (HDAC 4, 5, 7, 9) (39). To assess if butyrate impacts *IDO-1* expression through its HDACi property, we tested three HDACi targeting a wide range of HDAC. Two belonging to the hydroxamic acids family, structurally and metabolically unrelated to SCFAs: trichostatin A (TSA), Vorinostat (SAHA) and one belonging to the fatty acid family: sodium valproate (VAP) (39). The effect of butyrate on *IDO-1* expression was mimicked by the three HDACi tested suggesting that the *IDO-1* down-regulation observed with butyrate might be a consequence of its HDAC inhibitory properties (Figure 7A).

Regulation of gene transcription by butyrate involved a wide range of transcription factors. To delineate whether transcription factors targeted by butyrate could impact *IDO-1* expression, we analyzed the human *IDO-1* promoter sequence. Analysis revealed binding sites for several transcription factors implicated in butyrate-regulated gene expression, namely Specificity Protein-1 (SP1) binding GC-rich boxes, as well as AP1 and PPARγ responsive elements (Supplementary Table 2) (40–43). To delineate if butyrate affects *IDO-1* expression *via* SP1, we treated stimulated cells with mithramycin A that binds to GC-rich DNA sequences, thereby inhibiting SP1-dependent gene modulation (44). As shown in Figure 7A, incubation of butyrate or HDACi-stimulated cells with mithramycin did not impact on the *IDO-1* down-regulation, suggesting that SP1 was not involved in this process. As butyrate is a major activator of PPARγ-dependent gene activation, we also investigated its role in *IDO-1* down-regulation (43). Two specific PPARγ activators, pioglitazone and rosiglitazone, did not affect *IDO-1* expression, suggesting that the PPARγ responsive elements in *IDO-1* promoter might not be functional (Figure 7B). We further tested whether PPARγ was involved in the butyrate-dependent inhibition of *IDO-1* by using a specific PPARγ inhibitor (GW9662). The PPARγ inhibitor GW9662 did not impact on the butyrate-induced *IDO-1* down-regulation, confirming that the transcription factor PPARγ was not involved in this process (Figure 7B). Finally, the implication of AP1 motifs, present in *IDO-1* promoter was tested using an AP1 chemical inhibitor (SR11302). Pre-treatment with AP1 inhibitor did not significantly prevent the inhibition of *IDO-1* mediated by butyrate, suggesting that AP1 was not involved either (Figure 7C). Altogether, our findings suggest that butyrate down-regulates *IDO-1*-expression by a second mechanism involving its HDACi property, independently of the butyrate-targeted transcription factors AP1, PPARγ, and SP1.

## Discussion

The immune system is traditionally viewed as a highly elaborated defense system developed to fight intruders, especially rapidly evolving pathogens such as bacteria. However, accumulating studies highlight a widespread cooperation established between hosts and bacteria during millions of years that have shaped their own development (45). Intestinal commensal bacteria are crucial for the development and maintenance of a healthy immune system locally and have a homeostatic role beyond the gut, therefore contributing to the global well-being of their host. The particular abundance and combination of commensal bacteria may have dramatic and specific impacts on the host immune system through their intimate interaction with the host epithelium. Accordingly, the IECs play a central role in the dialogue established between the host and the microbiota by providing an active physical segregation of commensal bacteria and by initiating the first microbial-dependent signals. Indeed, IECs express receptors recognizing microbial motifs that activate downstream signaling cascades thus promoting the production of bactericidal peptides and the recruitment and activation of innate and adaptive immune cells notably by the production of effector proteins and enzymes (7, 10). Amongst the effector molecules modulating the immune responses produced by IECs, indoleamine 2,3-dioxygenase-1 (IDO-1) has an important role in the gut homeostasis (19, 46). However, whether human IECs express *IDO-1* and how bacteria control *IDO-1* expression in IECs is still unclear. Here, we show that human normal colonic IECs express *IDO-1* at the mRNA and protein level and that epithelial *IDO-1* is modulated by SCFAs, more specifically by butyrate. Indeed, we demonstrate herein that physiological concentrations of butyrate down-regulate *IDO-1* expression in HT-29 and Caco-2 reporter systems, but also at the mRNAs level in both the HT-29 cell line and in human primary colonic epithelial cells (30).

In the context of IFNγ stimulation, STAT1 is an essential mediator of *IDO-1* expression (31). Our results indicate that butyrate-treated IECs showed reduced STAT1 phosphorylation on the tyrosine 701, as described in other models (32, 33). However, our results indicate that the reduced amount of phosphorylated STAT1 observed with butyrate is a consequence of a butyrate-driven STAT1 protein level reduction. STAT1 diminution was not a result of an increase of nuclear translocation and we did not observe any transcriptional inhibition of *STAT1* expression, suggesting a post-transcriptional modification of STAT1. Many post-translational modifications of STAT1 such as SUMOylation and ubiquitination have been identified leading to STAT1 degradation and consequently modifying STAT1 protein levels in cells (47–49). Interestingly, butyrate has been described as a global enhancer of protein ubiquitination (32). We thus believe that combination of post-translational modifications of STAT1 might occur explaining its down-regulation by butyrate. The precise mechanism, and cellular actor, notably the implication of HDAC inhibition or GPRs implicated in STAT1 down-regulation need to be investigated further.

In addition to the butyrate-dependent down-regulation of STAT1 that impaired IFNγ-induced *IDO-1* expression, we demonstrated that STAT1 is dispensable for the basal *IDO-1* repression induced by butyrate suggesting that this SCFA repressed *IDO-1* expression by a second distinct mechanism. To decipher this STAT1-independent mechanism, we investigated the implication of butyrate specific G-protein coupled receptors (GPR41, GPR109a, and GPR43). However, by using agonists of these receptors and G protein subunit inhibitors, we showed that this mechanism was not implicated in the inhibition of *IDO-1* mediated by butyrate. SCFAs impact the host biological responses by the direct regulation of gene transcription by their properties of lysine deacetylase inhibitors that consequently favor acetylation of histones and transcription factors (4, 5). We showed that three HDAC inhibitors targeting a wide range of HDAC mimicked the effect of butyrate on *IDO-1* expression in un-stimulated cells suggesting that the *IDO-1* down-regulation observed was likely linked to the HDAC inhibitory properties of SCFAs. As regulation of gene transcription by HDACi involved many transcription factors, we reported, by analyzing the sequence of the *IDO-1* promoter, the presence of responsive elements of three transcription factors potentially targeted by butyrate: SP1, AP1, and PPARγ (40–43). However, by using specific inhibitors and agonists, we demonstrated that these three transcription factors were not involved in the STAT1-independent butyrate-driven inhibition of *IDO-1* expression.

Despite being limited to human cell-lines and primary IECs, our results highlighted a role of butyrate in *IDO-1* expression. However, *in vivo* studies are required to confirm these *in vitro* results and to precise the downstream effects of modulation of *IDO-1* in the colon. What would be the impact of *IDO-1* inhibition on human health is still an open question, as, depending on the disease context, its expression has positive or negative outcomes (14–16, 50). IDO-1 is highly expressed in human tumor cells and consequently creates an immunosuppressive microenvironment that has been associated with poor prognosis notably in colorectal cancer (19, 46). *IDO-1* expression is high in inflammatory bowel diseases notably in IECs and has often been positively associated with the severity of gastrointestinal diseases and inflammatory-induced colon tumorigenesis, with no causal implication (17–19, 46). However, *IDO-1*^−/−^ mice do not present any spontaneous colitis and its role in induced colitis models varies between studies according to the inducing agent and mouse strain used and probably the microbiota composition (51–54). IDO-1 regulates immune responses *via* the so-called “metabolic immune regulation” that suppresses the Th1 and Th17 differentiation and enhances the *de novo* differentiation of anti-inflammatory regulatory T cells (50). A recent study suggests that the role of IDO-1 in the regulation of the immune response is more complex as it repressed the production of IL10, a major anti-inflammatory cytokine (14). In line with this, recent studies suggest that IDO-1 expression have a detrimental role in aneurysm, atherosclerosis and obesity (14–16). Moreover, Laurans et al. demonstrate that IDO-1 activity enhanced chronic inflammation and intestinal permeability that consequently impacts on obesity outcomes (15). In addition, IDO-1 has been described as a main regulator of the intestinal B cell responses to commensal bacteria that drives microbiota composition and indirectly the microbiota-dependent barrier responses (55, 56). These studies demonstrate that intestinal IDO-1 expression might also shape gut microbiota with potent impact on host health. Altogether, these studies suggest that the role of IDO-1 in influencing gut inflammation is far more complex than expected, and might depend on the cell types expressing it. *IDO-1* down-regulation by microbiota-derived butyrate in IECs, as demonstrated here, could be crucial for the fine-tuning of *IDO-1* expression in healthy conditions and for the initiation of appropriate immune responses depending on the context: chronic inflammation, cancer, obesity or infections.

Here, we describe an important role for the SCFA butyrate in the regulation of *IDO-1* expression in IECs. Contrary to DCs where IDO-1 functions in diverse processes in health and disease have been well-documented, its role in IECs is still debated. We demonstrated here for the first time that butyrate represses *IDO-1* expression by two distinct mechanisms. First, butyrate treatment was able to reduce STAT1-dependent induction of *IDO-1*. In addition, we show that this reduction is correlated with the butyrate-driven decrease in STAT1 level. Second, butyrate regulation of *IDO-1* expression is independent of the IFNγ-signaling pathway and involves the HDAC inhibitory property of butyrate. As SCFAs are crucial for human physiology and health, our results strongly suggest that controlling *IDO-1* expression in IECs under steady state conditions can be part of the global mechanism of SCFAs to maintain immune homeostasis in the gut.

## Author Contributions

CM-G, NL conceived and designed the experiments. CM-G performed most of the experiments. PL, AJ, FB-C, LM, FL, and NL performed some experiments. CM-G, AJ, and NL analyzed the data. FR contributed materials. CM-G, NL wrote the paper. AJ, PL, FR, and HB edited and revised the manuscript.

### Conflict of Interest Statement

The authors declare that the research was conducted in the absence of any commercial or financial relationships that could be construed as a potential conflict of interest.
