# Supplementary material for: Butyrate Produced by Commensal Bacteria Down-Regulates Indolamine 2,3-Dioxygenase 1 (IDO-1) Expression via a Dual Mechanism in Human Intestinal Epithelial Cells
Source: Front Immunol. 2018 Dec 11;9:2838. doi: 10.3389/fimmu.2018.02838 (PMC6297836; doi:10.3389/fimmu.2018.02838)
Supplement: Supplementary file 1 [file Data_Sheet_1.PDF]

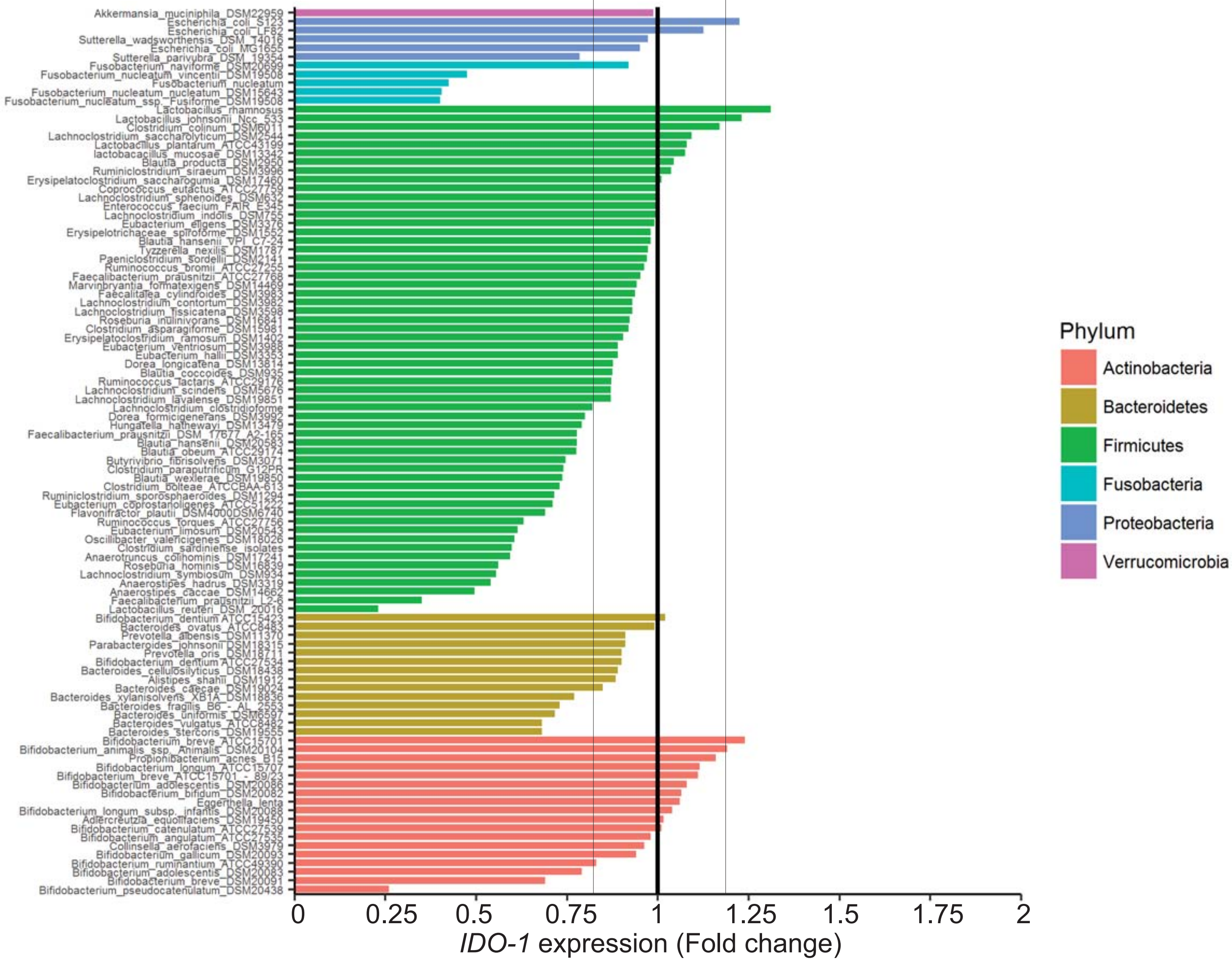

Supplementary Figure 1: Impact of commensal bacterial supernatants on *IDO-1* expression in HT-29. Culture supernatants of a wide range of cultivable commensal bacteria were applied on the HT-29-*IDO-1* reporter system (10% vol/vol). *IDO-1* expression was measured by luciferase activity and expressed as fold increase towards its control: bacterial growth medium used in each experiment. *IDO-1* expression profiles upper and lower the dash lines were considered as significantly changed. Actinobacteria in red, Bacteroidetes in yellow, Firmicutes in green, Fusobacteria in light blue, Proteobacteria in dark blue and Verrucomicrobia in pink.

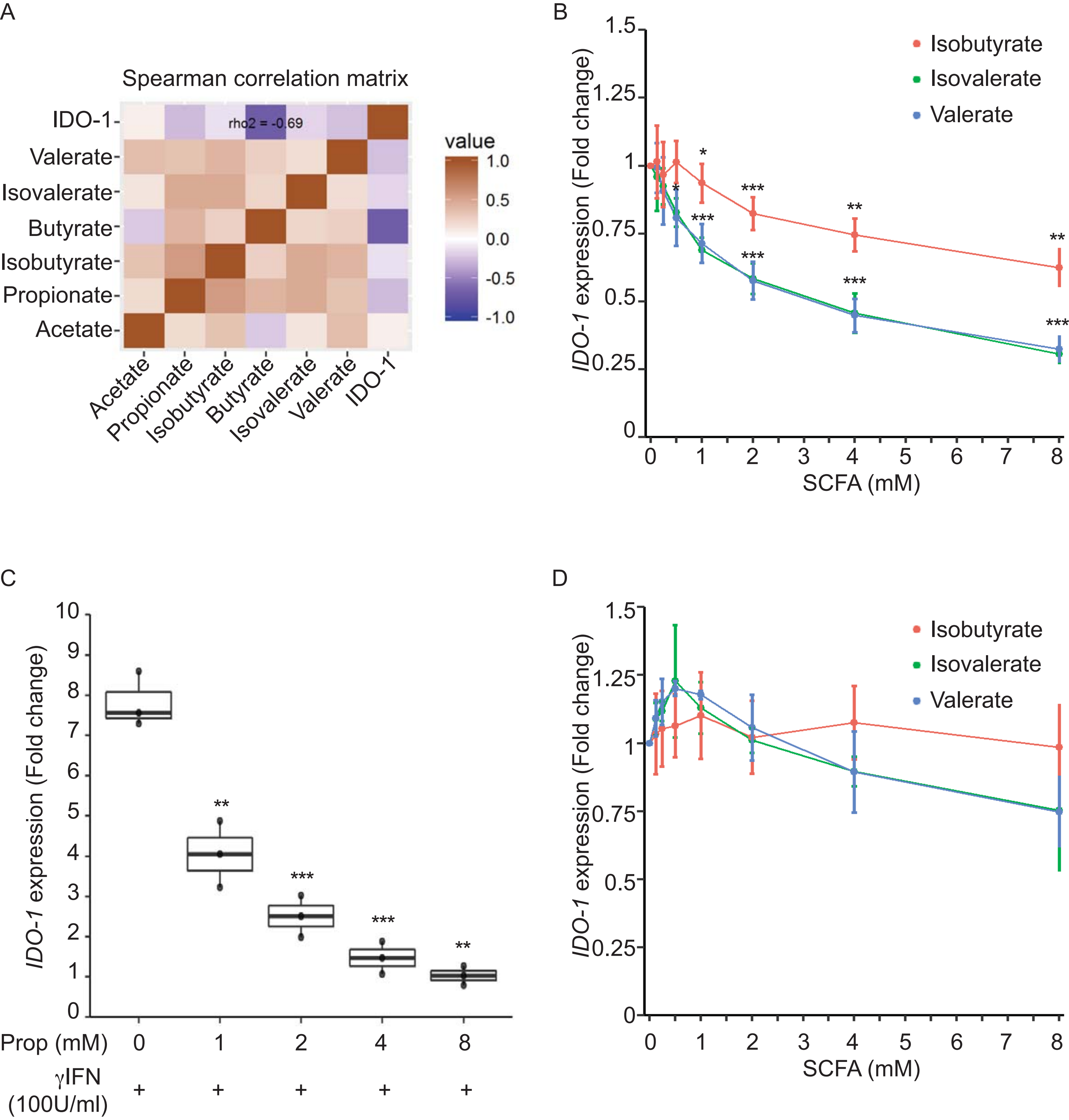

Supplementary Figure 2: Impact of SCFAs on *IDO-1* expression. (A) Spearman correlation matrix analyses showing the correlation between the SCFAs concentrations produced the commensal bacteria and the *IDO-1* expression. (B) HT-29-*IDO-1* reporter cells were incubated with a range of concentration of isobutyrate, isovalerate and valerate (0.5; 1; 2; 4; 8 mM). (C) HT-29-*IDO-1* reporter cells were incubated with IFNγ (100U/ml) and a range of concentration of propionate (1-8mM). (D) Caco2-*IDO-1* reporter cells were stimulated with a range of concentration of isobutyrate, isovalerate and valerate (0.5; 1; 2; 4; 8 mM). *IDO-1* expression was measured by luciferase activity and expressed as the mean ± SD fold change towards un-stimulated cells (B and D) or as the median ± quartiles of fold change towards un-stimulated cells (C). Data represented at least 3 independent experiments. P value: *P<0.05, **P<0.005, ***P<0.001.

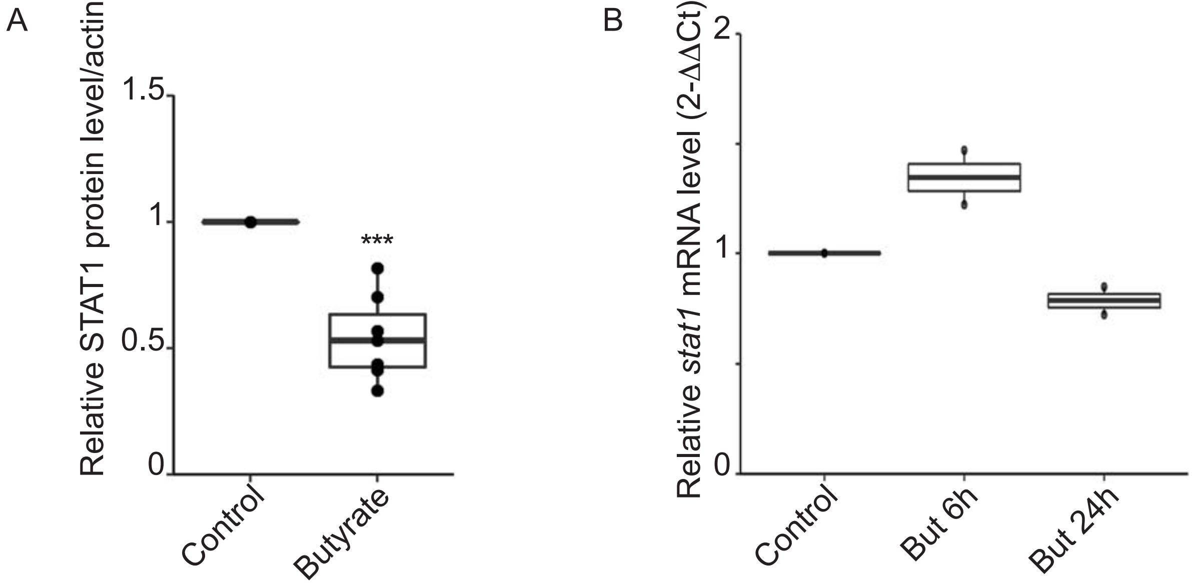

Supplementary Figure 3: (A) Densitometric quantifications of the total STAT1 protein levels from HT29 ± butyrate (But 2mM) after 24h of incubation. Results were from at least 3 independent experiments, normalised to Actin and expressed as fold change compared to un-stimulated cells (Control). (B) HT29 cells were incubated with butyrate for 6 or 24h and *STAT1* mRNA level was determined by RT-PCR, normalised by internal control (*GAPDH*) and express as 2-ΔCt toward non stimulated cells (N=3). P value: *P<0.05, **P<0.005, ***P<0.001.

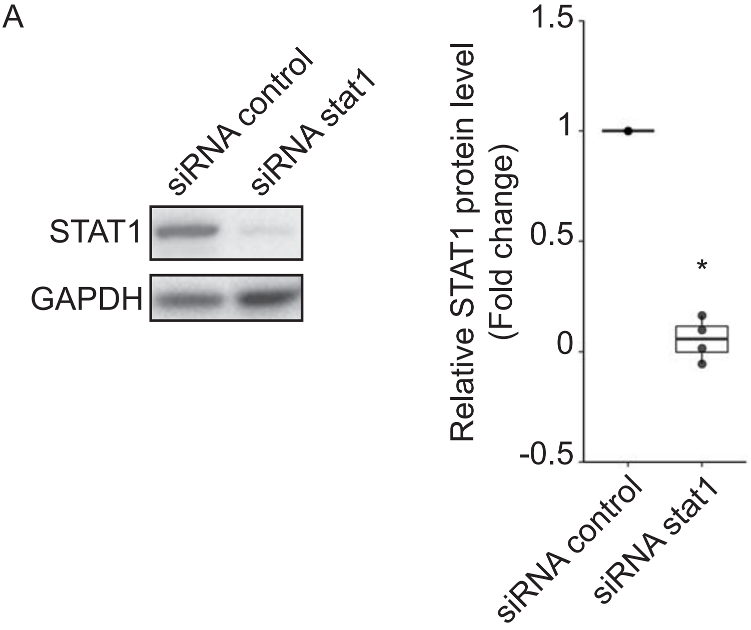

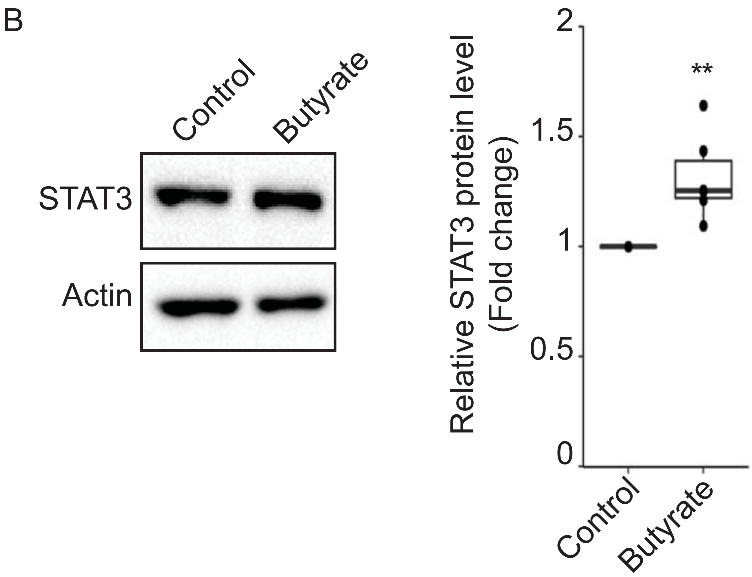

Supplementary Figure 4: STAT1 siRNA efficiency. HT29 cells were transfected with *STAT1* siRNA or control siRNA and protein extracted on day 7 following seeding. STAT1 and GAPDH protein level were determined by western blot (left panel). Densitometric quantifications of STAT1 was normalised to GAPDH and expressed as the median ± quartiles compared to siRNA control-treated cells (N=4, right panel). (B) HT-29 cells were treated with butyrate (But 2mM) for 24h before proteins extraction. The level of STAT3 and Actin were determined by western-blot on total protein extracted (left panel). Densitometric quantification of STAT3 protein levels were normalised to Actin and expressed as fold change compared to un-stimulated cell. Data are represented as median ± quartiles (N=6) (right panel). P value: *P<0.05, **P<0.005, ***P<0.001.

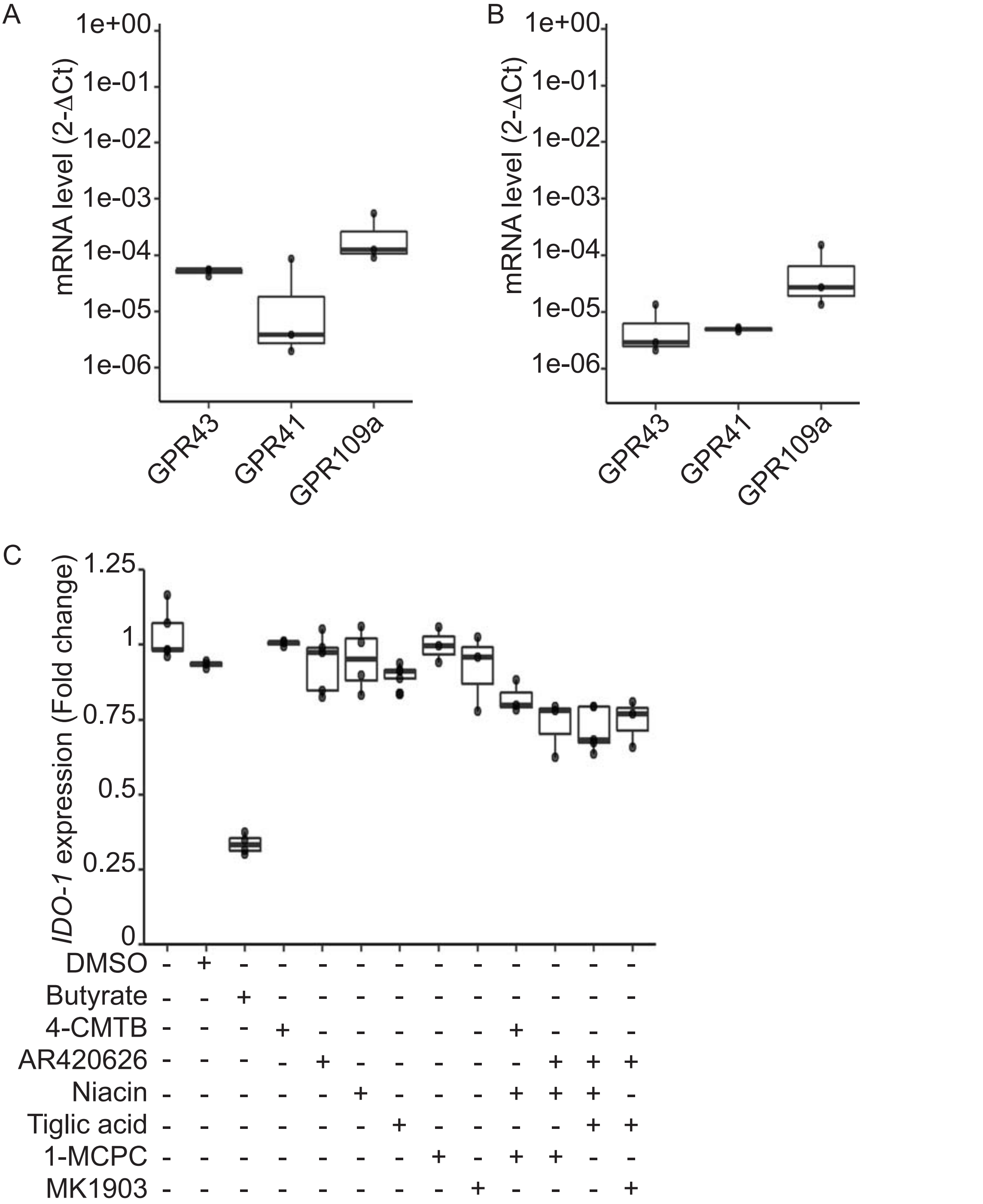

Supplementary Figure 5: Expression of the G-protein coupled receptors GPR41, GPR43 and GPR109a in HT-29 and Caco2. GPRs gene expression was determined by RT-PCR on mRNA extracted from HT-29 (A) and Caco2 (B) cells. Results are expressed as 2-ΔCt relative to *GAPDH* (N=3). (C) HT-29-*IDO-1* reporter cells were incubated for 24h with GPR alone or in combination: GPR41: AR420626 (1µM) and 1-MCPC (1mM); GPR43: 4-CMTB (1µM) and Tiglic acid (1mM); GPR109a: Niacin (1mM) and MK1903 (1µM) or with DMSO (vehicle) or butyrate (But 2mM). Data are represented as mean +/- standard deviation of at least 3 independent experiments. *IDO-1* expression was measured by luciferase activity and expressed as the median ± quartiles of fold change towards un-stimulated cells (N=3). P value: *P<0.05, **P<0.005, ***P<0.001.

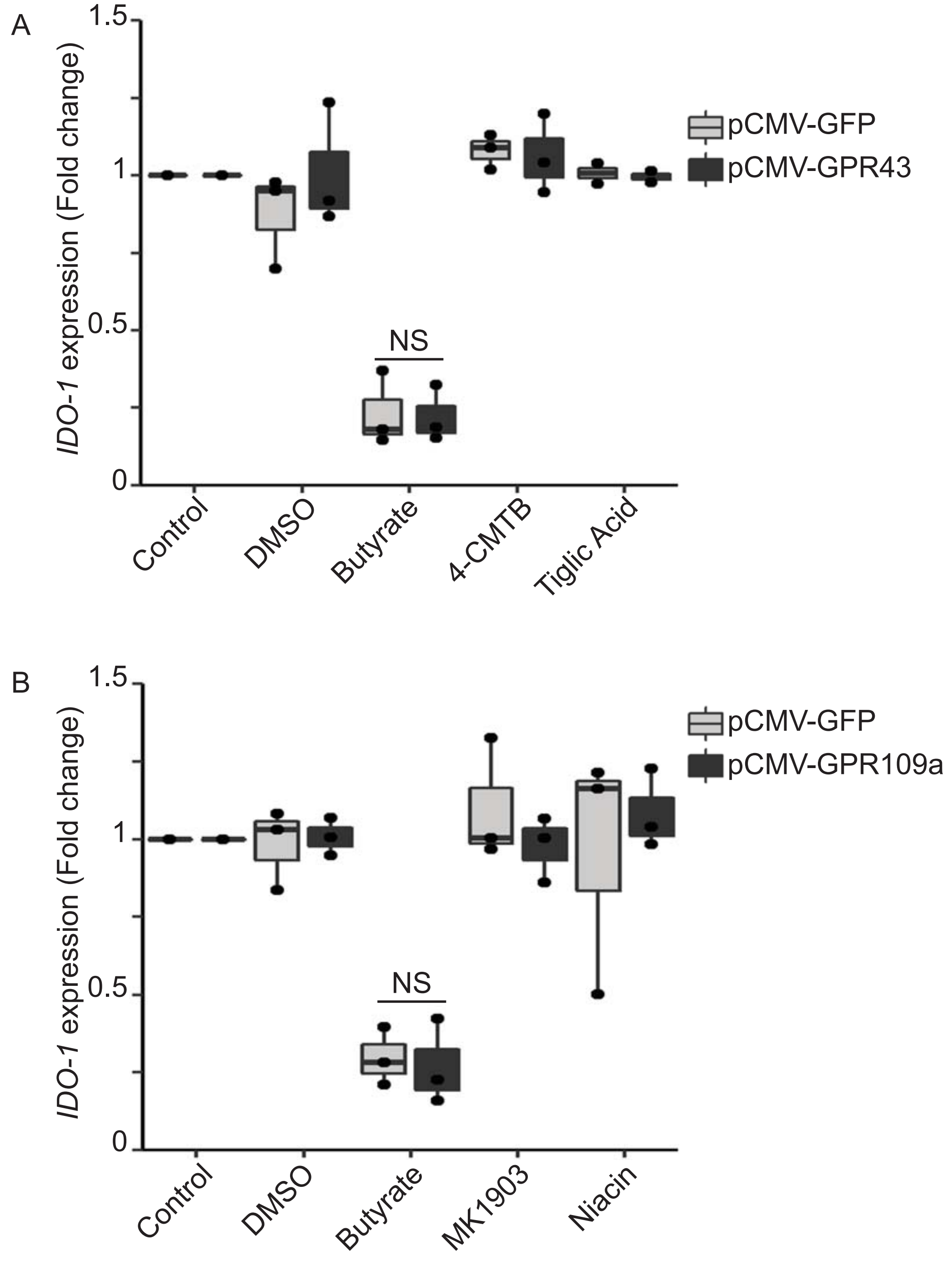

Supplementary Figure 6: GPR43 and GPR109a over-expression did not impact the butyrate-driven inhibition of *IDO-1* expression. HT-29-*IDO-1* reporter cells were transfected with pCMV-*GPR43* (A) and pCMV-*GPR109a* (B) prior to the incubation with 2mM butyrate or with the GPR agonists (GPR43 (A): 4-CMTB (1μM) and Tiglic acid (1mM) GPR109a (B): Niacine (1mM) and MK1903 (1μM)). *IDO-1* expression was measured by luciferase activity and expressed as the median ± quartiles of fold change towards unstimulated cells (N=3). P value: *P<0.05, **P<0.005, ***P<0.001.
