# Supplementary material for: Butyrate Produced by Commensal Bacteria Down-Regulates Indolamine 2,3-Dioxygenase 1 (IDO-1) Expression via a Dual Mechanism in Human Intestinal Epithelial Cells
Source: Front Immunol. 2018 Dec 11;9:2838. doi: 10.3389/fimmu.2018.02838 (PMC6297836; doi:10.3389/fimmu.2018.02838)
Supplement: Supplementary file 2 [file Data_Sheet_2.PDF]

Supplementary Table 1: Results of IDO-1 screening and SCFA measurements

						**Concentration in mM**						
**Bacterium**	**Aerobic**	**Phylum**	**Genus**	**OD**	**Medium**	**Acetate**	**Propionate**	**Isobutyrate**	**Butyrate**	**Isovalerate**	**Valerate**	**IDO activity**
Adlercreutzia equolifaciens DSM19450	NO	Actinobacteria	Adlercreutzia	0,22	Medium Wilkins-Chalgren	NA	NA	NA	NA	NA	NA	0,99
Adlercreutzia equolifaciens DSM19450	NO	Actinobacteria	Adlercreutzia	0,17	M104	NA	NA	NA	NA	NA	NA	1,04
Akkermansia muciniphila DSM22959	NO	Verrucomicrobia	Akkermansia	NA	LYBHI 4 mucin	5,090	3,250	-0,080	-0,090	0,000	0,000	1,15
Akkermansia muciniphila DSM22959	NO	Verrucomicrobia	Akkermansia	1,07	LYBHI 4 mucin	6,620	4,220	-0,020	-0,070	-0,030	0,000	0,94
Akkermansia muciniphila DSM22959	NO	Verrucomicrobia	Akkermansia	0,92	LYBHI 4 mucin	NA	NA	NA	NA	NA	NA	0,89
Akkermansia muciniphila DSM22959	NO	Verrucomicrobia	Akkermansia	1,41	LYBHI 4 mucin	NA	NA	NA	NA	NA	NA	0,97
Alistipes shahii DSM19121	NO	Bacteroidetes	Alistipes	0,37	LYBHI 4 RJ	NA	NA	NA	NA	NA	NA	1,06
Alistipes shahii DSM19121	NO	Bacteroidetes	Alistipes	1,76	LYBHI 4	NA	NA	NA	NA	NA	NA	0,76
Alistipes shahii DSM19121	NO	Bacteroidetes	Alistipes	0,85	M78	NA	NA	NA	NA	NA	NA	0,83
Allobaculum stercoricanis DSM13633	NO	Firmicutes	Allobaculum	0,26	LYBHI 4 RJ	NA	NA	NA	NA	NA	NA	1,01
Anaerostipes caccae DSM14662	NO	Firmicutes	Anaerostipes	1,58	M104	-4,330	0,000	0,000	14,460	0,000	0,000	0,54
Anaerostipes caccae DSM14662	NO	Firmicutes	Anaerostipes	0,89	M78 G	-5,130	2,620	0,000	15,930	-0,190	0,270	0,68
Anaerostipes caccae DSM14662	NO	Firmicutes	Anaerostipes	0,74	LYBHI 4 RJ	-10,100	-0,680	0,000	23,190	-0,040	0,110	0,47
Anaerostipes caccae DSM14662	NO	Firmicutes	Anaerostipes		LYBHI 4 RJ	NA	NA	NA	NA	NA	NA	0,41
Anaerostipes caccae DSM14662	NO	Firmicutes	Anaerostipes		M104	NA	NA	NA	NA	NA	NA	0,38
Anaerostipes hadrus DSM3319	NO	Firmicutes	Anaerostipes	1,94	M110	-2,050	-0,115	-0,045	17,525	0,248	-0,025	0,40
Anaerostipes hadrus DSM3319	NO	Firmicutes	Anaerostipes	NA	M78	-8,830	-0,085	-0,035	4,215	-0,043	-0,025	0,65
Anaerostipes hadrus DSM3319	NO	Firmicutes	Anaerostipes	0,60	M78	-8,080	-0,035	-0,015	4,015	0,068	-0,025	0,57
Anaerostipes hadrus DSM3319	NO	Firmicutes	Anaerostipes	1,92	M104	NA	NA	NA	NA	NA	NA	0,34
Anaerostipes hadrus DSM3319	NO	Firmicutes	Anaerostipes	1,17	LYBHI 4 RJ	NA	NA	NA	NA	NA	NA	0,42
Anaerostipes rhamnosivorans DSM26241	NO	Firmicutes	Anaerostipes	2,00	M104	NA	NA	NA	NA	NA	NA	0,28
Anaerostipes rhamnosivorans DSM26241	NO	Firmicutes	Anaerostipes	1,84	LYBHI 4 RJ	NA	NA	NA	NA	NA	NA	0,46
Anaerotruncus colihominis DSM17241	NO	Firmicutes	Anaerotruncus	NA	LYBHI 4 RJ	16,410	1,820	0,180	8,630	0,420	4,114	0,47
Anaerotruncus colihominis DSM17241	NO	Firmicutes	Anaerotruncus	NA	LYBHI 4 RJ	12,240	1,410	0,190	9,060	0,350	4,094	0,60
Anaerotruncus colihominis DSM17241	NO	Firmicutes	Anaerotruncus	NA	LYBHI 4 RJ	12,470	1,450	0,180	9,470	0,450	4,104	0,60
Anaerotruncus colihominis DSM17241	NO	Firmicutes	Anaerotruncus	1,05	LYBHI 4 RJ	6,940	0,000	0,000	8,620	0,080	3,894	0,57
Anaerotruncus colihominis DSM17241	NO	Firmicutes	Anaerotruncus	NA	LYBHI 4 RJ	14,840	1,750	0,180	8,600	0,370	4,084	0,55
Anaerotruncus colihominis DSM17241	NO	Firmicutes	Anaerotruncus	1,53	LYBHI 4 RJ	82,490	21,330	2,080	10,670	4,450	5,324	0,77
Atopobium parvulum DSM20469	NO	Actinobacteria	Atopobium	0,49	M104	NA	NA	NA	NA	NA	NA	1,26
Atopobium parvulum DSM20469	NO	Actinobacteria	Atopobium	0,50	Medium Wilkins-Chalgren	NA	NA	NA	NA	NA	NA	0,99
Atopobium parvulum DSM20469	NO	Actinobacteria	Atopobium	0,91	LYBHI 4 RJ	NA	NA	NA	NA	NA	NA	0,95
Bacteroides caccae DSM19024	NO	Bacteroidetes	Bacteroides	0,49	LYHBHI 1	9,690	9,990	0,090	-0,220	1,850	0,000	0,74
Bacteroides caccae DSM19024	NO	Bacteroidetes	Bacteroides	1,39	Medium Wilkins-Chalgren	NA	NA	NA	NA	NA	NA	1,04
Bacteroides caccae DSM19024	NO	Bacteroidetes	Bacteroides	1,81	LYBHI 4 RJ	NA	NA	NA	NA	NA	NA	0,77
Bacteroides caccae DSM19024	NO	Bacteroidetes	Bacteroides	0,95	M110	NA	NA	NA	NA	NA	NA	0,84
Bacteroides cellulosilyticus DSM14838	NO	Bacteroidetes	Bacteroides	1,48	LYBHI 4 RJ	NA	NA	NA	NA	NA	NA	1,00
Bacteroides cellulosilyticus DSM14838	NO	Bacteroidetes	Bacteroides	0,26	M78	NA	NA	NA	NA	NA	NA	0,78
Bacteroides fragilis DSM2151	NO	Bacteroidetes	Bacteroides	0,50	LYHBHI 1	10,500	13,830	0,320	-0,180	3,390	0,000	0,73
Bacteroides ovatus DSM1896	NO	Bacteroidetes	Bacteroides	0,68	M104	9,690	0,030	-1,050	-0,240	0,070	0,000	0,99
Bacteroides spp D8	NO	Bacteroidetes	Bacteroides	0,74	M104	10,570	0,090	-1,830	-0,240	0,030	0,000	1,03
Bacteroides stercoris DSM19555	NO	Bacteroidetes	Bacteroides	0,87	M78	NA	NA	NA	NA	NA	NA	0,68
Bacteroides thetaiotaomicron DSM2079	NO	Bacteroidetes	Bacteroides	0,77	M104	9,920	0,050	-1,790	-0,240	0,060	0,000	1,16
Bacteroides thetaiotaomicron DSM2079	NO	Bacteroidetes	Bacteroides	1,70	M78	NA	NA	NA	NA	NA	NA	0,89
Bacteroides thetaiotaomicron DSM2079	NO	Bacteroidetes	Bacteroides	1,42	Medium Wilkins-Chalgren	NA	NA	NA	NA	NA	NA	0,77
Bacteroides thetaiotaomicron DSM2079	NO	Bacteroidetes	Bacteroides	0,30	M110	NA	NA	NA	NA	NA	NA	0,97
Bacteroides uniformis DSM6597	NO	Bacteroidetes	Bacteroides	1,09	M104	9,770	46,690	-1,330	-0,190	0,280	0,000	0,46
Bacteroides uniformis DSM6597	NO	Bacteroidetes	Bacteroides	0,70	LYBHI 4 RJ	NA	NA	NA	NA	NA	NA	0,83
Bacteroides uniformis DSM6597	NO	Bacteroidetes	Bacteroides	NA	LYBHI 4	NA	NA	NA	NA	NA	NA	0,86
Bacteroides vulgatus DSM1447	NO	Bacteroidetes	Bacteroides	0,44	LYHBHI 1	13,730	10,070	0,260	-0,230	2,460	0,000	0,68
Bacteroides xylanisolvens DSM18836	NO	Bacteroidetes	Bacteroides	1,06	LYBHI 4	NA	NA	NA	NA	NA	NA	0,80
Bacteroides xylanisolvens DSM18836	NO	Bacteroidetes	Bacteroides	0,94	LYBHI 4 RJ	NA	NA	NA	NA	NA	NA	0,74
Bifidobacterium adolescentis DSM20083	NO	Actinobacteria	Bifidobacterium	0,88	M58	73,420	0,000	-1,030	0,020	0,000	0,000	0,79
Bifidobacterium adolescentis DSM20086	NO	Actinobacteria	Bifidobacterium	1,48	LYBHI 4	NA	NA	NA	NA	NA	NA	1,29
Bifidobacterium adolescentis DSM20086	NO	Actinobacteria	Bifidobacterium	2,00	M58	NA	NA	NA	NA	NA	NA	0,87
Bifidobacterium angulatum DSM20098	NO	Actinobacteria	Bifidobacterium	1,07	M58	69,210	0,000	-1,100	-0,010	0,000	0,000	0,98
Bifidobacterium animalis subsp animalis DSM20104	NO	Actinobacteria	Bifidobacterium	0,68	M58	49,850	0,000	-0,360	0,060	0,000	0,000	1,19
Bifidobacterium animalis subsp animalis DSM20104	NO	Actinobacteria	Bifidobacterium	NA	M58	NA	NA	NA	NA	NA	NA	1,19
Bifidobacterium bifidum DSM20082	NO	Actinobacteria	Bifidobacterium	0,79	M58	69,390	0,000	-0,900	-0,010	0,000	0,000	1,04
Bifidobacterium bifidum DSM20082	NO	Actinobacteria	Bifidobacterium	NA	M58	NA	NA	NA	NA	NA	NA	1,09
Bifidobacterium breve ATCC15701	NO	Actinobacteria	Bifidobacterium	NA	M58	NA	NA	NA	NA	NA	NA	1,24
Bifidobacterium breve ATCC15701	NO	Actinobacteria	Bifidobacterium	0,31	M104	15,610	0,000	-1,400	-0,180	0,000	0,000	1,11
Bifidobacterium breve DSM20091	NO	Actinobacteria	Bifidobacterium	0,52	M104	1,130	12,520	-1,020	-0,190	0,240	0,000	0,69
Bifidobacterium breve DSM20091	NO	Actinobacteria	Bifidobacterium	1,33	M58	NA	NA	NA	NA	NA	NA	1,00
Bifidobacterium catenulatum DSM16992	NO	Actinobacteria	Bifidobacterium	1,07	M58	62,140	0,000	-0,810	-0,010	0,000	0,000	1,01
Bifidobacterium choerinum DSM20434	NO	Actinobacteria	Bifidobacterium	0,97	M58	69,050	0,000	-1,040	0,000	0,000	0,000	1,03

Bifidobacterium choerinum DSM20434	NO	Actinobacteria	Bifidobacterium	NA	M58	NA	NA	NA	NA	NA	NA	1,13
Bifidobacterium dentium DSM20084	NO	Actinobacteria	Bifidobacterium	0,88	M104	48,720	0,000	-1,840	-0,220	0,000	0,000	1,02
Bifidobacterium dentium DSM20084	NO	Actinobacteria	Bifidobacterium	NA	M58	NA	NA	NA	NA	NA	NA	0,80
Bifidobacterium dentium DSM20436	NO	Actinobacteria	Bifidobacterium	0,80	M104	42,870	0,000	-1,660	-0,180	0,000	0,000	0,90
Bifidobacterium gallicum DSM20093	NO	Actinobacteria	Bifidobacterium	0,58	M58	51,140	0,000	-0,570	0,000	0,000	0,000	0,94
Bifidobacterium gallicum DSM20093	NO	Actinobacteria	Bifidobacterium	1,22	M58	NA	NA	NA	NA	NA	NA	1,02
Bifidobacterium longum subsp infantis DSM20088	NO	Actinobacteria	Bifidobacterium	0,76	M58	49,770	0,000	-1,010	-0,010	0,000	0,000	1,04
Bifidobacterium longum subsp infantis DSM20088	NO	Actinobacteria	Bifidobacterium	1,32	M58	NA	NA	NA	NA	NA	NA	0,99
Bifidobacterium pseudocatenulatum DSM20438	NO	Actinobacteria	Bifidobacterium	1,27	M58	71,020	0,000	-1,070	-0,020	0,000	0,000	0,26
Bifidobacterium ruminantium DSM6489	NO	Actinobacteria	Bifidobacterium	1,08	M58	61,090	0,000	-1,150	-0,010	0,000	0,000	0,83
Bifidobacterium longum subsp longum DSM20219	NO	Actinobacteria	Bifidobacterium	0,90	M58	65,040	0,000	-0,840	-0,020	0,000	0,000	1,02
Bifidobacterium longum subsp longum DSM20219	NO	Actinobacteria	Bifidobacterium	NA	M58	NA	NA	NA	NA	NA	NA	1,21
Blautia coccoides DSM935	NO	Firmicutes	Blautia	1,69	LYBHI 1	39,370	-0,140	-0,060	-0,080	-0,090	0,897	0,78
Blautia coccoides DSM935	NO	Firmicutes	Blautia	1,94	LYBHI 1	32,580	-0,130	-0,060	-0,060	-0,080	0,897	0,76
Blautia coccoides DSM935	NO	Firmicutes	Blautia	1,88	LYBHI 1 mucin	40,220	-0,130	0,020	-0,050	-0,080	0,897	0,86
Blautia coccoides DSM935	NO	Firmicutes	Blautia	1,91	LYBHI 4 RJ	46,250	0,500	0,020	0,200	0,020	0,010	0,59
Blautia coccoides DSM935	NO	Firmicutes	Blautia	1,78	M110	34,690	-0,365	-0,075	-0,415	0,138	3,842	0,96
Blautia coccoides DSM935	NO	Firmicutes	Blautia	0,79	M78	21,000	-0,415	-0,075	-0,415	-0,053	3,842	1,04
Blautia coccoides DSM935	NO	Firmicutes	Blautia	1,25	M78	20,320	-0,395	-0,075	-0,415	-0,083	3,842	0,92
Blautia coccoides DSM935	NO	Firmicutes	Blautia	1,49	M78	21,720	-0,365	-0,075	-0,275	-0,073	3,842	0,92
Blautia coccoides DSM935	NO	Firmicutes	Blautia	0,79	M78	37,140	-0,375	-0,015	-0,225	-0,133	3,842	1,04
Blautia gnavus B07	NO	Firmicutes	Blautia	1,45	LYBHI 4	28,060	3,240	0,360	1,820	0,650	0,350	0,64
Blautia gnavus B07	NO	Firmicutes	Blautia	0,89	LYBHI 1	9,600	-0,070	0,010	-0,030	-0,060	0,000	0,82
Blautia gnavus B07	NO	Firmicutes	Blautia	1,21	LYBHI 4	26,570	3,450	0,370	1,870	0,250	0,330	0,66
Blautia gnavus B09	NO	Firmicutes	Blautia	1,44	LYBHI 4	24,540	3,440	0,370	1,850	0,690	0,330	0,71
Blautia gnavus B94	NO	Firmicutes	Blautia	1,56	LYBHI 4	26,170	3,350	0,360	1,810	0,680	0,320	0,67
Blautia gnavus B94	NO	Firmicutes	Blautia	1,12	LYBHI 1	12,380	0,160	0,030	0,100	0,080	0,000	0,82
Blautia gnavus B94	NO	Firmicutes	Blautia	NA	LYBHI 4	29,000	3,900	0,420	2,090	0,330	0,370	0,72
Blautia gnavus FRE1	NO	Firmicutes	Blautia	1,63	LYBHI 4	11,830	0,020	0,000	0,020	0,040	0,000	1,02
Blautia gnavus FRE1	NO	Firmicutes	Blautia	1,91	LYBHI 4	13,960	0,000	-0,010	0,010	-0,400	0,000	0,97
Blautia gnavus FRE1	NO	Firmicutes	Blautia	1,79	LYBHI 4 RJ	14,570	0,330	0,020	-0,050	0,010	-0,120	0,97
Blautia gnavus V62	NO	Firmicutes	Blautia	1,86	LYBHI 4	15,450	37,140	0,350	1,700	0,830	0,320	0,53
Blautia gnavus V62	NO	Firmicutes	Blautia	1,62	LYBHI 4	10,540	29,830	0,370	1,720	0,340	0,270	0,64
Blautia gnavus V62	NO	Firmicutes	Blautia	1,62	LYBHI 4	10,410	29,210	0,340	1,630	0,310	0,350	0,64
Blautia gnavus V62	NO	Firmicutes	Blautia	NA	LYBHI 4	6,600	0,080	-0,020	-0,070	0,400	0,000	0,99
Blautia gnavus V96	NO	Firmicutes	Blautia	1,96	LYBHI 4	19,570	41,630	0,380	1,750	0,970	0,290	0,53
Blautia gnavus V96	NO	Firmicutes	Blautia	NA	LYBHI 1	3,150	3,000	-0,010	-0,060	0,040	0,000	0,77
Blautia gnavus V96	NO	Firmicutes	Blautia	1,55	LYBHI 4	15,460	34,580	0,350	1,680	0,440	0,290	0,60
Blautia hansenii DSM20583	NO	Firmicutes	Blautia	1,54	M104	10,610	-0,170	-0,020	-0,180	-0,030	0,000	0,88
Blautia hansenii DSM20583	NO	Firmicutes	Blautia	1,54	M104	79,580	19,820	1,810	10,060	3,960	1,670	0,66
Blautia hansenii DSM20583	NO	Firmicutes	Blautia	1,59	M104	3,770	0,000	0,000	0,000	0,000	0,000	0,79
Blautia hansenii DSM20583	NO	Firmicutes	Blautia	1,30	LYBHI 4	9,190	0,000	0,000	0,020	0,000	0,000	0,98
Blautia hydrogenotrophica DSM10507	NO	Firmicutes	Blautia	1,39	LYBHI 4	21,130	0,050	1,080	0,030	1,990	0,000	0,87
Blautia hydrogenotrophica DSM10507	NO	Firmicutes	Blautia	NA	LYBHI 4 RJ	30,810	-0,120	4,570	-0,120	9,290	-0,120	0,85
Blautia hydrogenotrophica DSM10507	NO	Firmicutes	Blautia	NA	LYBHI 4 RJ	16,140	0,780	0,520	0,320	1,290	-0,120	0,79
Blautia hydrogenotrophica DSM10507	NO	Firmicutes	Blautia	1,50	LYBHI 4 RJ	30,350	-0,080	0,260	-0,010	0,450	0,030	1,05
Blautia hydrogenotrophica DSM10507	NO	Firmicutes	Blautia	0,60	M78	3,150	0,145	0,545	-0,005	1,358	-0,025	0,83
Blautia hydrogenotrophica DSM10507	NO	Firmicutes	Blautia	1,58	M104	14,660	0,000	0,000	0,000	0,000	0,000	0,80
Blautia obeum DSM2538	NO	Firmicutes	Blautia	1,47	LYBHI 4	24,750	-0,020	-0,010	0,030	0,060	0,000	0,88
Blautia obeum DSM2538	NO	Firmicutes	Blautia	1,79	LYBHI 4	27,780	0,130	0,020	0,150	0,040	0,000	0,80
Blautia obeum DSM2538	NO	Firmicutes	Blautia	1,95	LYBHI 4	28,490	0,380	0,070	0,280	0,160	0,000	0,73
Blautia obeum DSM2538	NO	Firmicutes	Blautia	2,00	LYBHI 4 RJ	28,620	0,380	0,060	0,160	0,110	0,030	0,68
Blautia obeum DSM2538	NO	Firmicutes	Blautia	1,81	LYBHI 4 RJ	31,730	0,200	0,030	0,020	0,070	-0,010	0,89
Blautia obeum DSM2538	NO	Firmicutes	Blautia	1,78	LYBHI 4	28,060	0,540	0,080	0,390	0,230	0,000	0,67
Blautia producta DSM2950	NO	Firmicutes	Blautia	0,57	M78	-4,610	-0,375	-0,075	-0,415	-0,023	-0,025	1,08
Blautia producta DSM2950	NO	Firmicutes	Blautia	1,03	M104	0,190	-0,150	-0,080	-0,180	0,020	0,000	1,05
Blautia producta DSM2950	NO	Firmicutes	Blautia	1,94	M104	2,580	-0,160	-0,070	-0,180	0,040	0,000	1,01
Blautia producta DSM2950	NO	Firmicutes	Blautia	1,31	LYBHI 4	3,210	-0,180	-0,080	-0,110	-0,030	0,000	1,05
Blautia producta DSM2950	NO	Firmicutes	Blautia	1,38	LYBHI 4 RJ	3,020	-0,060	-0,020	-0,230	-0,040	-0,120	1,03
Blautia wexlerae DSM19850	NO	Firmicutes	Blautia	0,27	M104	15,880	3,520	0,370	1,810	0,690	0,350	0,73
Blautia wexlerae DSM19850	NO	Firmicutes	Blautia	0,41	M78	11,210	3,535	0,395	1,725	0,688	0,375	0,62
Blautia wexlerae DSM19850	NO	Firmicutes	Blautia	0,39	M78	-4,420	0,185	0,025	0,015	0,128	-0,025	0,86
Butyrivibrio fibrisolvens DSM3071	NO	Firmicutes	Butyrivibrio	0,40	LYBHI 4 RJ	104,860	30,280	3,200	15,290	6,000	2,840	1,00
Butyrivibrio fibrisolvens DSM3071	NO	Firmicutes	Butyrivibrio	0,40	LYBHI 4 RJ	104,860	30,280	3,200	15,290	6,000	2,840	0,31
Butyrivibrio fibrisolvens DSM3071	NO	Firmicutes	Butyrivibrio	0,79	LYBHI 4 RJ	4,110	0,390	0,000	8,300	0,000	0,090	0,64
Butyrivibrio fibrisolvens DSM3071	NO	Firmicutes	Butyrivibrio	0,34	LYBHI 4	1,890	0,000	0,000	4,510	0,030	0,000	0,81
Butyrivibrio fibrisolvens DSM3071	NO	Firmicutes	Butyrivibrio	0,66	LYBHI 2	1,640	0,000	-0,070	-0,050	0,020	0,000	0,97

Clostridium asparagiforme DSM15981	NO	Firmicutes	Lachnoclostridium	0,30	M104	2,060	-0,070	-0,020	-0,030	0,160	0,000	1,01
Clostridium asparagiforme DSM15981	NO	Firmicutes	Lachnoclostridium	1,63	M104	16,290	0,000	0,000	0,000	0,000	0,000	0,88
Clostridium asparagiforme DSM15981	NO	Firmicutes	Lachnoclostridium	NA	M104	NA	NA	NA	NA	NA	NA	0,89
Clostridium asparagiforme DSM15981	NO	Firmicutes	Lachnoclostridium	1,14	M104	NA	NA	NA	NA	NA	NA	0,85
Clostridium asparagiforme DSM15981	NO	Firmicutes	Lachnoclostridium	0,32	M104	NA	NA	NA	NA	NA	NA	0,90
Clostridium asparagiforme DSM15981	NO	Firmicutes	Lachnoclostridium	0,51	M78	-4,100	-0,335	-0,075	-0,415	-0,073	-0,025	1,04
Clostridium asparagiforme DSM15981	NO	Firmicutes	Lachnoclostridium	2,16	LYBHI 4 RJ	18,010	-0,120	0,000	-0,100	-0,010	0,000	0,88
Clostridium asparagiforme DSM15981	NO	Firmicutes	Lachnoclostridium	1,94	LYBHI 4 RJ	18,830	-0,080	0,000	0,000	-0,090	0,000	0,87
Clostridium asparagiforme DSM15981	NO	Firmicutes	Lachnoclostridium	1,88	LYBHI 4	17,560	0,000	0,000	0,040	0,070	0,000	0,85
Clostridium asparagiforme DSM15981	NO	Firmicutes	Lachnoclostridium	2,00	Medium Wilkins-Chalgren	22,480	0,000	0,000	0,000	0,000	0,000	1,03
Clostridium bolteae DSM15670	NO	Firmicutes	Lachnoclostridium	1,24	M104	16,160	-0,110	-0,080	-0,180	-0,030	0,000	0,84
Clostridium bolteae DSM15670	NO	Firmicutes	Lachnoclostridium	1,67	M104	17,080	-0,170	-0,080	-0,180	-0,030	0,000	0,51
Clostridium bolteae DSM15670	NO	Firmicutes	Lachnoclostridium	1,61	LYBHI 4 RJ	21,900	0,370	0,000	0,170	-0,010	0,030	0,84
Clostridium colinum DSM6011	NO	Firmicutes	Tyzzerella	0,70	M78	2,880	-0,415	-0,075	-0,415	-0,073	-0,025	1,19
Clostridium colinum DSM6011	NO	Firmicutes	Tyzzerella	0,39	M78	-1,220	-0,355	-0,075	-0,415	-0,033	-0,025	1,15
Clostridium paraputrificum G12PR - X73445	NO	Firmicutes	Clostridium	0,20	LYHBHI 1	7,750	0,000	0,150	3,710	0,000	0,000	0,74
Clostridium phytofermentans DSM18823	NO	Firmicutes	Clostridium	1,08	M110	NA	NA	NA	NA	NA	NA	1,14
Clostridium phytofermentans DSM18823	NO	Firmicutes	Clostridium	1,08	M78	NA	NA	NA	NA	NA	NA	1,40
Clostridium phytofermentans DSM18823	NO	Firmicutes	Clostridium	0,46	LYBHI 4 RJ	NA	NA	NA	NA	NA	NA	1,75
Clostridium phytofermentans DSM18823	NO	Firmicutes	Clostridium	0,5	Medium Wilkins-Chalgren	NA	NA	NA	NA	NA	NA	1,50
Clostridium sardiniense C3pr	NO	Firmicutes	Clostridium	0,31	M78	2,920	0,160	0,020	7,720	0,000	0,000	0,63
Clostridium sardiniense C7pr	NO	Firmicutes	Clostridium	0,39	M78	5,540	0,430	0,000	15,230	0,000	0,000	0,52
Clostridium sardiniense C7pr	NO	Firmicutes	Clostridium	1,52	M78	NA	NA	NA	NA	NA	NA	0,58
Clostridium sardiniense C9pr	NO	Firmicutes	Clostridium	0,29	M78	7,060	0,440	0,000	7,920	0,000	0,000	0,70
Clostridium sardiniense D12pr	NO	Firmicutes	Clostridium	0,40	M78	2,530	0,050	0,000	6,150	0,000	0,000	0,54
Clostridium sardiniense D12pr	NO	Firmicutes	Clostridium	1,73	M78	NA	NA	NA	NA	NA	NA	0,58
Clostridium sardiniense D7pr	NO	Firmicutes	Clostridium	0,57	M78	6,530	0,000	-0,100	-0,200	0,000	0,000	0,55
Clostridium sardiniense I11 pr	NO	Firmicutes	Clostridium	0,34	M78	2,170	0,000	7,970	13,220	0,000	0,000	0,65
Clostridium sardiniense I11 pr	NO	Firmicutes	Clostridium	0,99	M78	NA	NA	NA	NA	NA	NA	0,63
Collinsella aerofaciens DSM3979	NO	Actinobacteria	Collinsella	NA	Medium Wilkins-Chalgren	9,570	0,000	0,110	0,010	0,030	0,000	1,02
Collinsella aerofaciens DSM3979	NO	Actinobacteria	Collinsella	0,19	LYHBHI 1	3,960	0,000	-0,150	-0,170	0,000	0,000	0,89
Collinsella aerofaciens DSM3979	NO	Actinobacteria	Collinsella	1,41	M104	NA	NA	NA	NA	NA	NA	0,94
Collinsella aerofaciens DSM3979	NO	Actinobacteria	Collinsella	1,30	Medium Wilkins-Chalgren	NA	NA	NA	NA	NA	NA	1,01
Collinsella aerofaciens DSM3979	NO	Actinobacteria	Collinsella	2,00	LYBHI 4 RJ	NA	NA	NA	NA	NA	NA	0,95
Coprococcus eutactus ATCC27759	NO	Firmicutes	Coprococcus	0,41	M78	0,960	1,355	-0,015	0,135	0,178	-0,025	1,20
Coprococcus eutactus ATCC27759	NO	Firmicutes	Coprococcus	0,97	M110	-4,490	12,375	-0,035	-0,215	0,488	-0,025	0,85
Coprococcus eutactus ATCC27759	NO	Firmicutes	Coprococcus	0,41	M78	-5,760	0,385	-0,035	-0,105	-0,033	-0,025	0,96
Dorea formicigenerans DSM3992	NO	Firmicutes	Dorea	0,86	M110	-1,820	0,555	-0,045	6,525	0,138	-0,025	1,20
Dorea formicigenerans DSM3992	NO	Firmicutes	Dorea	1,45	M78	7,260	19,225	-0,075	4,645	0,548	-0,025	0,72
Dorea formicigenerans DSM3992	NO	Firmicutes	Dorea	0,56	LYBHI 1	2,440	0,650	-0,080	2,390	0,000	0,000	0,48
Dorea longicatena DSM13814	NO	Firmicutes	Dorea	2,00	M104	12,910	0,000	0,000	0,000	0,000	0,000	0,79
Dorea longicatena DSM13814	NO	Firmicutes	Dorea	1,37	LYBHI 4 RJ	12,480	-0,120	0,000	-0,050	-0,050	0,050	0,86
Dorea longicatena DSM13814	NO	Firmicutes	Dorea	1,58	LYBHI 4	11,210	0,000	0,000	0,020	0,000	0,000	0,98
Edwardsiella tarda ATCC23685	YES	Proteobacteria	Edwardsiella	NA	M3	NA	NA	NA	NA	NA	NA	1,31
Edwardsiella tarda ATCC23685	YES	Proteobacteria	Edwardsiella	NA	M3	NA	NA	NA	NA	NA	NA	1,24
Edwardsiella tarda ATCC23685	YES	Proteobacteria	Edwardsiella	NA	M104	NA	NA	NA	NA	NA	NA	0,88
Edwardsiella tarda ATCC23685	YES	Proteobacteria	Edwardsiella	0,35	M1	NA	NA	NA	NA	NA	NA	1,26
Edwardsiella tarda ATCC23685	YES	Proteobacteria	Edwardsiella	0,25	M1	NA	NA	NA	NA	NA	NA	1,44
Edwardsiella tarda ATCC23685	YES	Proteobacteria	Edwardsiella	0,60	M1	NA	NA	NA	NA	NA	NA	1,59
Edwardsiella tarda ATCC23685	YES	Proteobacteria	Edwardsiella	1,54	LB	NA	NA	NA	NA	NA	NA	1,57
Edwardsiella tarda ATCC23685	YES	Proteobacteria	Edwardsiella	1,18	M104	NA	NA	NA	NA	NA	NA	2,16
Edwardsiella tarda DSM30052	YES	Proteobacteria	Edwardsiella	0,85	M1	NA	NA	NA	NA	NA	NA	1,46
Edwardsiella tarda DSM30052	YES	Proteobacteria	Edwardsiella	0,29	M1	NA	NA	NA	NA	NA	NA	1,11
Edwardsiella tarda DSM30052	YES	Proteobacteria	Edwardsiella	1,46	M1	NA	NA	NA	NA	NA	NA	1,04
Edwardsiella tarda DSM30052	YES	Proteobacteria	Edwardsiella	0,44	M1	NA	NA	NA	NA	NA	NA	0,95
Edwardsiella tarda DSM30052	YES	Proteobacteria	Edwardsiella	0,36	M1	NA	NA	NA	NA	NA	NA	0,97
Edwardsiella tarda DSM30052	YES	Proteobacteria	Edwardsiella	3,16	LB	NA	NA	NA	NA	NA	NA	0,94
Edwardsiella tarda DSM30052	YES	Proteobacteria	Edwardsiella	0,82	LB	NA	NA	NA	NA	NA	NA	0,88
Edwardsiella tarda DSM30052	YES	Proteobacteria	Edwardsiella	0,73	LB	NA	NA	NA	NA	NA	NA	0,98
Edwardsiella tarda DSM30052	YES	Proteobacteria	Edwardsiella	1,34	LYBHI 1	NA	NA	NA	NA	NA	NA	1,25
Edwardsiella tarda DSM30052	YES	Proteobacteria	Edwardsiella	0,30	M1	NA	NA	NA	NA	NA	NA	1,37
Edwardsiella tarda DSM30052	YES	Proteobacteria	Edwardsiella	0,25	M1	NA	NA	NA	NA	NA	NA	1,28
Edwardsiella tarda DSM30052	YES	Proteobacteria	Edwardsiella	0,65	M1	NA	NA	NA	NA	NA	NA	1,51
Edwardsiella tarda DSM30052	YES	Proteobacteria	Edwardsiella	1,33	LB	NA	NA	NA	NA	NA	NA	1,50
Edwardsiella tarda DSM30052	YES	Proteobacteria	Edwardsiella	1,03	M104	NA	NA	NA	NA	NA	NA	2,16
Eggerthella lenta DSM2243	NO	Actinobacteria	Eggerthella	NA	Medium Wilkins-Chalgren	1,710	0,000	0,000	0,090	0,120	0,000	1,12
Eggerthella lenta DSM2243	NO	Actinobacteria	Eggerthella	NA	Medium Wilkins-Chalgren	1,200	0,000	0,000	0,000	0,110	0,000	1,00

Enterococcus faecium FAIR E345	YES	Firmicutes	Enterococcus	NA	BHI aerobic	2,500	0,000	0,010	0,010	-0,010	0,000	1,00
Erysipelatoclostridium ramosum DSM1402	NO	Firmicutes	Erysipelatoclostridium	1,19	LYBHI 4 RJ	23,020	0,050	0,000	10,44	0,02	0,01	0,83
Erysipelatoclostridium ramosum DSM1402	NO	Firmicutes	Erysipelatoclostridium	1,57	LYBHI 4 RJ	16,790	0,280	0,000	-0,10	-0,01	-0,12	0,74
Erysipelatoclostridium ramosum DSM1402	NO	Firmicutes	Erysipelatoclostridium	1,78	LYBHI 4	14,580	0,150	0,010	0,06	0,06	0,00	1,07
Erysipelatoclostridium ramosum DSM1402	NO	Firmicutes	Erysipelatoclostridium	1,43	LYBHI 1	14,290	0,230	0,040	0,14	-0,02	0,00	0,98
Erysipelatoclostridium saccharogumia DSM17460	NO	Firmicutes	Erysipelatoclostridium	1,22	M104	3,830	0,000	0,000	0,00	0,00	0,00	1,16
Erysipelatoclostridium saccharogumia DSM17460	NO	Firmicutes	Erysipelatoclostridium	0,58	M104	6,840	0,000	0,000	0,00	0,00	0,00	0,86
Erysipelotrichaceae spiroforme DSM1552	NO	Firmicutes	Erysipelatoclostridium	0,77	M110	8,400	0,000	0,000	0,07	-0,33	0,00	0,86
Erysipelotrichaceae spiroforme DSM1552	NO	Firmicutes	Erysipelatoclostridium	0,97	LYBHI 4 RJ	73,610	5,630	0,440	3,83	0,51	0,33	0,97
Erysipelotrichaceae spiroforme DSM1552	NO	Firmicutes	Erysipelatoclostridium	0,99	LYBHI 4	8,420	0,080	0,000	0,17	0,04	0,00	1,11
Escherichia coli LF82	YES	Proteobacteria	Escherichia	NA	LB	NA	NA	NA	NA	NA	NA	1,16
Escherichia coli LF82	YES	Proteobacteria	Escherichia	NA	LB	NA	NA	NA	NA	NA	NA	1,09
Escherichia coli MG1655	YES	Proteobacteria	Escherichia	NA	LB	NA	NA	NA	NA	NA	NA	0,95
Escherichia coli S123	YES	Proteobacteria	Escherichia	NA	LB	NA	NA	NA	NA	NA	NA	1,16
Escherichia coli S123	YES	Proteobacteria	Escherichia	NA	LB	NA	NA	NA	NA	NA	NA	1,29
Eubacterium coprostanoligenes ATCC51222	NO	Firmicutes	Eubacterium	NA	LYBHI 4 Cholesterol phos	NA	NA	NA	NA	NA	NA	0,71
Eubacterium eligens DSM3376	NO	Firmicutes	Eubacterium	0,49	M436	-0,510	0,000	0,650	-1,00	-0,91	0,00	1,02
Eubacterium eligens DSM3376	NO	Firmicutes	Eubacterium	1,30	M104	3,750	0,000	0,000	0,00	0,01	0,00	1,08
Eubacterium eligens DSM3376	NO	Firmicutes	Eubacterium	1,03	LYBHI 4 RJ	-3,710	-1,320	1,000	-0,86	1,65	0,00	0,93
Eubacterium eligens DSM3376	NO	Firmicutes	Eubacterium	0,82	LYBHI 4	NA	NA	NA	NA	NA	NA	0,93
Eubacterium hallii DSM3353	NO	Firmicutes	Eubacterium	0,38	M78	-5,960	-0,205	-0,045	-0,245	-0,013	-0,025	0,89
Eubacterium limosum DSM20543	NO	Firmicutes	Eubacterium	NA	LYBHI 3	13,870	0,040	0,420	6,200	0,410	0,110	0,43
Eubacterium limosum DSM20543	NO	Firmicutes	Eubacterium	NA	M104	23,460	0,050	-0,020	12,310	0,010	0,000	0,41
Eubacterium limosum DSM20543	NO	Firmicutes	Eubacterium	2,22	M104	19,200	-0,050	-0,040	13,460	0,020	0,110	1,00
Eubacterium rectale ATCC33656 - CIP105953	NO	Firmicutes	Eubacterium	0,23	LYHBHI 1	0,100	4,640	-0,150	-0,160	0,000	0,000	0,74
Eubacterium ventriosum DSM3988	NO	Firmicutes	Eubacterium	0,27	M110	-0,690	0,515	-0,005	0,125	0,168	-0,025	1,09
Eubacterium ventriosum DSM3988	NO	Firmicutes	Eubacterium	NA	M110 K1	8,010	29,720	0,000	0,000	0,820	0,000	0,71
Eubacterium ventriosum DSM3988	NO	Firmicutes	Eubacterium	0,96	LYBHI 4 RJ	-1,280	4,470	0,000	-0,100	-0,020	0,020	0,87
Faecalibacterium prausnitzii	NO	Firmicutes	Faecalibacterium	1,18	LYBHI 2	-1,210	-0,080	-0,020	7,780	-0,010	0,000	0,63
Faecalibacterium prausnitzii ATCC27768	NO	Firmicutes	Faecalibacterium	0,61	Medium Wilkins-Chalgren	NA	NA	NA	NA	NA	NA	0,96
Faecalibacterium prausnitzii ATCC27768	NO	Firmicutes	Faecalibacterium	0,85	LYBHI 4	NA	NA	NA	NA	NA	NA	0,89
Faecalibacterium prausnitzii ATCC27768	NO	Firmicutes	Faecalibacterium	0,42	LYBHI 4 RJ	NA	NA	NA	NA	NA	NA	1,03
Faecalibacterium prausnitzii ATCC27768	NO	Firmicutes	Faecalibacterium	0,64	M110	NA	NA	NA	NA	NA	NA	0,93
Faecalibacterium prausnitzii DSM 17677 A2-165	NO	Firmicutes	Faecalibacterium	1,66	LYBHI Fretter	-2,740	0,000	0,000	7,480	0,010	0,000	0,74
Faecalibacterium prausnitzii DSM 17677 A2-165	NO	Firmicutes	Faecalibacterium	1,70	LYBHI 4	18,640	-0,030	-0,020	-0,030	-0,460	0,000	1,11
Faecalibacterium prausnitzii DSM 17677 A2-165	NO	Firmicutes	Faecalibacterium	0,90	LYBHI 4	-0,830	-0,020	-0,020	8,020	-0,060	0,000	0,54
Faecalibacterium prausnitzii DSM 17677 A2-165	NO	Firmicutes	Faecalibacterium	1,60	LYBHI 4	18,130	0,110	-0,020	-0,050	0,350	0,000	1,10
Faecalibacterium prausnitzii DSM 17677 A2-165	NO	Firmicutes	Faecalibacterium	0,59	LYBHI 4	-5,520	0,000	0,000	8,630	0,020	0,000	0,74
Faecalibacterium prausnitzii DSM 17677 A2-165	NO	Firmicutes	Faecalibacterium	0,36	LYBHI 4	-0,490	1,340	0,000	15,310	0,140	0,080	0,76
Faecalibacterium prausnitzii DSM 17677 A2-165	NO	Firmicutes	Faecalibacterium	NA	LYBHI 4 RJ	-9,900	-1,320	0,000	6,110	-0,110	0,000	0,62
Faecalibacterium prausnitzii DSM 17677 A2-165	NO	Firmicutes	Faecalibacterium	1,35	LYBHI 4 RJ	NA	NA	NA	NA	NA	NA	0,61
Faecalibacterium prausnitzii L2-6	NO	Firmicutes	Faecalibacterium	NA	LYBHI 4	-5,220	0,000	0,000	6,880	0,000	0,000	0,42
Faecalibacterium prausnitzii L2-6	NO	Firmicutes	Faecalibacterium	0,91	LYBHI 4	8,350	0,050	0,000	0,000	0,010	0,000	0,28
Faecalicatena contorta DSM3982	NO	Firmicutes	Lachnoclostridium	1,92	M110	-0,460	0,425	-0,005	0,135	0,208	-0,025	0,89
Faecalicatena contorta DSM3982	NO	Firmicutes	Lachnoclostridium	1,23	M78	15,700	-0,245	-0,055	-0,415	0,218	-0,025	1,02
Faecalicatena contorta DSM3982	NO	Firmicutes	Lachnoclostridium	2,52	M110 K1	18,900	0,000	0,170	0,000	-0,440	0,000	0,93
Faecalicatena contorta DSM3982	NO	Firmicutes	Lachnoclostridium	2,02	LYBHI 4 RJ	17,130	-0,120	0,000	-0,030	-0,090	0,000	0,88
Faecalicatena fissicatena DSM3598	NO	Firmicutes	Lachnoclostridium	0,26	M104 C	3,580	0,000	0,000	0,000	0,000	0,000	0,96
Faecalicatena fissicatena DSM3598	NO	Firmicutes	Lachnoclostridium	1,30	Medium Wilkins-Chalgren	18,170	0,000	0,000	0,000	0,020	0,000	0,99
Faecalicatena fissicatena DSM3598	NO	Firmicutes	Lachnoclostridium	1,30	LYBHI 4 RJ	11,420	-0,150	0,000	-0,060	-0,040	0,040	0,84
Faecalitalea cylindroides DSM3983	NO	Firmicutes	Faecalitalea	2,21	M110	1,990	0,000	0,000	0,000	-0,440	0,000	0,92
Faecalitalea cylindroides DSM3983	NO	Firmicutes	Faecalitalea	2,21	M110	1,990	0,000	0,000	0,000	-0,440	0,000	0,92
Faecalitalea cylindroides DSM3983	NO	Firmicutes	Faecalitalea	2,51	M104	-0,050	0,000	0,000	0,000	0,000	0,000	0,82
Faecalitalea cylindroides DSM3983	NO	Firmicutes	Faecalitalea	1,80	LYBHI 4 RJ	2,210	-0,080	0,000	-0,040	0,050	0,000	1,00
Faecalitalea cylindroides DSM3983	NO	Firmicutes	Faecalitalea	0,2	LYBHI 4 RJ	NA	NA	NA	NA	NA	NA	1,03
Faecalitalea cylindroides DSM3983	NO	Firmicutes	Faecalitalea	0,35	M78	NA	NA	NA	NA	NA	NA	0,93
Flavonifractor plautii DSM6740	NO	Firmicutes	Flavonifractor	0,86	M110	-1,360	0,795	-0,045	4,305	0,238	-0,025	0,70
Flavonifractor plautii DSM6740	NO	Firmicutes	Flavonifractor	1,47	M78	20,350	28,380	0,050	5,970	1,280	0,130	0,43
Flavonifractor plautii DSM6740	NO	Firmicutes	Flavonifractor	0,50	M78	-2,290	0,665	-0,045	2,915	-0,043	-0,025	0,76
Flavonifractor plautii DSM6740	NO	Firmicutes	Flavonifractor	NA	M110	4,470	9,865	-0,075	5,435	0,628	-0,025	0,61
Flavonifractor plautii DSM6740	NO	Firmicutes	Flavonifractor	NA	LYBHI 4 RJ	15,990	13,280	0,010	10,660	0,110	0,050	0,95
Fusicatenibacter saccharivorans DSM 26062	NO	Firmicutes	Fusicatenibacter	1,71	M104	NA	NA	NA	NA	NA	NA	1,14
Fusicatenibacter saccharivorans DSM 26062	NO	Firmicutes	Fusicatenibacter	1,72	LYBHI 4 RJ	NA	NA	NA	NA	NA	NA	0,94
Fusicatenibacter saccharivorans DSM 26063	NO	Firmicutes	Fusicatenibacter	1,62	M104	NA	NA	NA	NA	NA	NA	1,14
Fusicatenibacter saccharivorans DSM 26063	NO	Firmicutes	Fusicatenibacter	0,90	LYBHI 4 RJ	NA	NA	NA	NA	NA	NA	0,92
Fusobacterium naviforme DSM20699	NO	Fusobacteria	Fusobacterium	NA	M104	7,450	1,810	-1,610	23,660	0,000	0,000	0,92
Fusobacterium nucleatum polymorphum DSM20482	NO	Fusobacteria	Fusobacterium	NA	M104	7,800	2,060	0,000	16,650	0,030	0,010	0,56

Fusobacterium nucleatum polymorphum DSM20482	NO	Fusobacteria	Fusobacterium	NA	M104	5,470	2,620	0,000	18,360	0,100	0,000	0,29
Fusobacterium nucleatum subsp nucleatum DSM15643	NO	Fusobacteria	Fusobacterium	1,53	M104	NA	NA	NA	NA	NA	NA	0,36
Fusobacterium nucleatum subsp nucleatum DSM15643	NO	Fusobacteria	Fusobacterium	1,47	LYBHI 4 RJ	NA	NA	NA	NA	NA	NA	0,45
Fusobacterium nucleatum subsp fusiforme DSM19508	NO	Fusobacteria	Fusobacterium	0,40	M78	28,700	0,900	0,070	24,340	0,000	0,000	0,40
Fusobacterium nucleatum subsp fusiforme DSM19508	NO	Fusobacteria	Fusobacterium	0,40	M78	28,700	0,900	0,070	24,340	0,000	0,000	0,40
Fusobacterium nucleatum subsp vincentii DSM19508	NO	Fusobacteria	Fusobacterium	1,46	M104	NA	NA	NA	NA	NA	NA	0,47
Fusobacterium nucleatum subsp vincentii DSM19508	NO	Fusobacteria	Fusobacterium	1,61	LYBHI 4 RJ	NA	NA	NA	NA	NA	NA	0,48
Hungatella hathewayi DSM13479	NO	Firmicutes	Hungatella	1,64	LYBHI 4 RJ	20,840	1,690	0,080	0,870	-0,040	-0,020	0,80
Hungatella hathewayi DSM13479	NO	Firmicutes	Hungatella	0,59	M104	3,370	-0,170	-0,030	-0,040	-0,030	0,000	1,03
Hungatella hathewayi DSM13479	NO	Firmicutes	Hungatella	0,37	M104	6,340	-0,020	-0,030	-0,030	-0,010	0,000	1,03
Hungatella hathewayi DSM13479	NO	Firmicutes	Hungatella	1,43	M104	NA	NA	NA	NA	NA	NA	0,30
Lachnoclostridium clostridioforme	NO	Firmicutes	Lachnoclostridium	1,20	M412	11,200	0,000	0,000	0,000	0,000	0,000	0,89
Lachnoclostridium clostridioforme	NO	Firmicutes	Lachnoclostridium	0,34	LYBHI 4 RJ	19,230	-0,060	0,000	0,030	-0,060	0,000	0,75
Lachnoclostridium indolis DSM755	NO	Firmicutes	Lachnoclostridium	0,90	M78	3,660	-0,335	-0,035	-0,235	0,007	-0,025	0,97
Lachnoclostridium indolis DSM755	NO	Firmicutes	Lachnoclostridium	1,98	M110	15,340	-0,265	-0,035	0,305	0,278	-0,025	0,88
Lachnoclostridium indolis DSM755	NO	Firmicutes	Lachnoclostridium	1,18	M78	0,020	-0,305	-0,045	-0,255	-0,053	-0,025	1,07
Lachnoclostridium indolis DSM755	NO	Firmicutes	Lachnoclostridium	1,72	LYBHI 4 RJ	16,070	0,510	0,030	0,250	0,070	0,000	0,94
Lachnoclostridium indolis DSM755	NO	Firmicutes	Lachnoclostridium	2	LYBHI 4 RJ	NA	NA	NA	NA	NA	NA	1,06
Lachnoclostridium indolis DSM755	NO	Firmicutes	Lachnoclostridium	1,56	M110	NA	NA	NA	NA	NA	NA	1,05
Lachnoclostridium lavalense DSM19851	NO	Firmicutes	Lachnoclostridium	1,49	M215c	11,750	0,000	0,000	0,000	-0,030	0,000	0,87
Lachnoclostridium lavalense DSM19851	NO	Firmicutes	Lachnoclostridium	1,70	LYBHI 4 RJ	12,830	-0,050	0,000	0,010	-0,030	0,040	0,87
Lachnoclostridium saccharolyticum DSM2544	NO	Firmicutes	Lachnoclostridium	1,99	M110	8,230	-0,295	-0,075	-0,275	0,218	-0,025	0,95
Lachnoclostridium saccharolyticum DSM2544	NO	Firmicutes	Lachnoclostridium	1,20	M110	9,270	-0,315	-0,075	-0,295	0,498	-0,025	1,27
Lachnoclostridium saccharolyticum DSM2544	NO	Firmicutes	Lachnoclostridium	1,49	M78	1,510	-0,325	-0,035	-0,415	-0,173	-0,025	1,06
Lachnoclostridium scindens DSM5676	NO	Firmicutes	Lachnoclostridium	1,38	LYBHI 4 RJ	20,240	-0,100	0,000	0,010	-0,010	0,040	0,87
Lachnoclostridium sphenoides DSM632	NO	Firmicutes	Lachnoclostridium	1,98	M110	10,910	-0,51	-0,08	-0,07	0,15	-0,03	1,00
Lachnoclostridium symbiosum DSM934	NO	Firmicutes	Lachnoclostridium	1,69	M110	11,630	-0,15	-0,08	6,11	0,24	-0,03	0,61
Lachnoclostridium symbiosum DSM934	NO	Firmicutes	Lachnoclostridium	1,23	M110	22,710	0,16	-0,04	14,70	0,29	-0,03	0,56
Lachnoclostridium symbiosum DSM934	NO	Firmicutes	Lachnoclostridium	0,64	M78	3,220	-0,25	-0,04	6,99	-0,04	-0,03	0,72
Lachnoclostridium symbiosum DSM934	NO	Firmicutes	Lachnoclostridium	0,52	M78	6,050	-0,30	-0,08	8,15	-0,05	-0,03	0,40
Lachnoclostridium symbiosum DSM934	NO	Firmicutes	Lachnoclostridium	1,14	LYBHI 4 RJ	24,650	0,11	0,02	10,73	0,06	-0,12	0,48
Lactobacacillus mucosae DSM13342	NO	Firmicutes	Lactobacillus	1,22	LYBHI 4 RJ	NA	NA	NA	NA	NA	NA	1,19
Lactobacacillus mucosae DSM13342	NO	Firmicutes	Lactobacillus	0,86	LYBHI 4	NA	NA	NA	NA	NA	NA	0,96
Lactobacillus acidophilus DSM 20079	NO	Firmicutes	Lactobacillus	1,71	M58	NA	NA	NA	NA	NA	NA	1,29
Lactobacillus casei Z313	NO	Firmicutes	Lactobacillus	2,00	M58	NA	NA	NA	NA	NA	NA	0,87
Lactobacillus johnsonii Ncc 533	NO	Firmicutes	Lactobacillus	1,72	M58	NA	NA	NA	NA	NA	NA	1,23
Lactobacillus paracasei Z763	NO	Firmicutes	Lactobacillus	2,00	M58	NA	NA	NA	NA	NA	NA	0,90
Lactobacillus plantarum ATCC43199	NO	Firmicutes	Lactobacillus	0,67	M58	NA	NA	NA	NA	NA	NA	1,30
Lactobacillus plantarum ATCC43199	NO	Firmicutes	Lactobacillus	0,71	M58	NA	NA	NA	NA	NA	NA	0,86
Lactobacillus reuteri DSM 20016	NO	Firmicutes	Lactobacillus	1,22	M58	NA	NA	NA	NA	NA	NA	0,45
Lactobacillus reuteri DSM 20016	NO	Firmicutes	Lactobacillus	NA	M104	NA	NA	NA	NA	NA	NA	0,01
Lactobacillus rhamnosus	NO	Firmicutes	Lactobacillus	1,57	M58	NA	NA	NA	NA	NA	NA	1,31
Lactobacillus rhamnosus Z712	NO	Firmicutes	Lactobacillus	2,00	M58	NA	NA	NA	NA	NA	NA	1,03
Marvinbryantia formatexigens DSM14469	NO	Firmicutes	Marvinbryantia	0,59	LYBHI 4	NA	NA	NA	NA	NA	NA	0,86
Marvinbryantia formatexigens DSM14469	NO	Firmicutes	Marvinbryantia	0,31	LYBHI 4 RJ	98,560	26,27	3,03	14,22	5,68	2,68	1,03
Marvinbryantia formatexigens DSM14469	NO	Firmicutes	Marvinbryantia	0,67	LYBHI 4 RJ	NA	NA	NA	NA	NA	NA	1,05
Marvinbryantia formatexigens DSM14469	NO	Firmicutes	Marvinbryantia	0,28	M110	NA	NA	NA	NA	NA	NA	0,86
Marvinbryantia formatexigens DSM14469	NO	Firmicutes	Marvinbryantia	0,31	LYBHI 4	NA	NA	NA	NA	NA	NA	0,91
Oscillibacter valericigenes DSM18026	NO	Firmicutes	Oscillibacter	0,75	M104	6,010	0,94	0,17	11,28	0,70	0,00	0,60
Oscillibacter valericigenes DSM18026	NO	Firmicutes	Oscillibacter	1,04	LYBHI 4 RJ	-5,080	-0,120	0,510	10,890	1,210	0,000	0,61
Oscillibacter valericigenes DSM18026	NO	Firmicutes	Oscillibacter	1,05	LYBHI 4 RJ	NA	NA	NA	NA	NA	NA	0,44
Oscillibacter valericigenes DSM18026	NO	Firmicutes	Oscillibacter	1,11	M104	NA	NA	NA	NA	NA	NA	0,39
Paeniclostridium sordellii DSM2141	NO	Firmicutes	Peptoclostridium	0,14	LYBHI 4	4,380	0,000	-0,130	0,050	0,000	0,000	0,97
Parabacteroides distasonis ATCC8503	NO	Bacteroidetes	Parabacteroides	0,67	LYHBHI 1	9,520	32,770	0,830	-0,220	5,570	0,000	0,53
Parabacteroides johnsonii DSM18315	NO	Bacteroidetes	Parabacteroides	0,60	M104	6,995	2,260	-1,790	-0,240	0,350	0,000	0,91
Prevotella albensis DSM11370	NO	Bacteroidetes	Prevotella	1,12	M78	NA	NA	NA	NA	NA	NA	0,81
Prevotella albensis DSM11370	NO	Bacteroidetes	Prevotella	1,32	Medium Wilkins-Chalgren	NA	NA	NA	NA	NA	NA	1,01
Prevotella bivia DSM20514	NO	Bacteroidetes	Prevotella	1,73	M104	NA	NA	NA	NA	NA	NA	NA
Prevotella copri DSM18205	NO	Bacteroidetes	Prevotella	0,44	M104	21,740	0,000	-1,210	-0,190	0,000	0,000	0,81
Prevotella copri DSM18205	NO	Bacteroidetes	Prevotella	0,51	LYBHI 4 RJ	NA	NA	NA	NA	NA	NA	1,14
Prevotella copri DSM18205	NO	Bacteroidetes	Prevotella	0,89	LYBHI 4	NA	NA	NA	NA	NA	NA	1,03
Prevotella copri DSM18205	NO	Bacteroidetes	Prevotella	0,73	M104	NA	NA	NA	NA	NA	NA	1,22
Prevotella copri DSM18205	NO	Bacteroidetes	Prevotella	1,17	M104	NA	NA	NA	NA	NA	NA	0,67
Prevotella oris DSM18711	NO	Bacteroidetes	Prevotella	1,66	M104	NA	NA	NA	NA	NA	NA	0,90
Propionibacterium acnes B15	NO	Actinobacteria	Propionibacterium	0,84	M104	-0,170	0,690	-1,490	-0,160	0,020	0,000	1,16
Roseburia faecis DSM16840	NO	Firmicutes	Roseburia	NA	LYBHI 4 RJ	-7,850	0,110	-0,010	20,390	0,040	0,160	0,46
Roseburia faecis DSM16840	NO	Firmicutes	Roseburia	0,85	LYBHI 4 RJ	-6,740	0,220	-0,010	17,990	0,030	0,110	0,54

Roseburia faecis DSM16840	NO	Firmicutes	Roseburia	0,25	M58	-15,360	-0,190	-0,040	11,250	-0,030	0,000	0,52
Roseburia hominis DSM16839	NO	Firmicutes	Roseburia	1,40	LYBHI 4 RJ	-2,850	0,240	-0,010	15,250	0,050	0,080	0,45
Roseburia hominis DSM16839	NO	Firmicutes	Roseburia	NA	LYBHI 4 RJ	-2,290	0,350	-0,010	15,190	0,060	0,080	0,48
Roseburia hominis DSM16839	NO	Firmicutes	Roseburia	0,72	M58	-11,720	-0,140	-0,030	11,620	-0,010	0,000	0,57
Roseburia hominis DSM16839	NO	Firmicutes	Roseburia	0,68	M58	-0,690	0,000	-0,280	10,370	0,000	0,000	0,77
Roseburia intestinalis DSM14610	NO	Firmicutes	Roseburia	0,39	LYBHI 4	-2,290	-0,010	-0,030	11,690	-0,470	0,000	0,49
Roseburia intestinalis DSM14610	NO	Firmicutes	Roseburia	0,39	LYBHI 4	-2,290	-0,010	-0,030	11,690	-0,470	0,000	0,63
Roseburia intestinalis DSM14610	NO	Firmicutes	Roseburia	0,68	LYBHI 4	-2,510	0,010	-0,030	13,230	-0,020	0,000	0,47
Roseburia intestinalis DSM14610	NO	Firmicutes	Roseburia	0,68	LYBHI 4	-2,510	0,010	-0,030	13,230	-0,020	0,000	0,54
Roseburia intestinalis DSM14610	NO	Firmicutes	Roseburia	0,86	M104	-1,100	2,850	0,410	30,230	0,890	0,680	0,61
Roseburia intestinalis DSM14610	NO	Firmicutes	Roseburia	0,86	M104	-1,100	2,850	0,410	30,230	0,890	0,680	0,47
Roseburia intestinalis DSM14610	NO	Firmicutes	Roseburia	0,80	M104	-1,840	0,030	-0,080	8,030	-0,030	0,000	0,47
Roseburia intestinalis DSM14610	NO	Firmicutes	Roseburia	0,80	M104	-1,840	0,030	-0,080	8,030	-0,030	0,000	NA
Roseburia intestinalis DSM14610	NO	Firmicutes	Roseburia	NA	M58	-15,270	-0,170	-0,010	21,480	-0,040	0,000	1,10
Roseburia intestinalis DSM14610	NO	Firmicutes	Roseburia	NA	M58	-15,270	-0,170	-0,010	21,480	-0,040	0,000	0,50
Roseburia intestinalis DSM14610	NO	Firmicutes	Roseburia	0,22	M58	-1,880	0,000	-0,030	12,990	0,000	0,000	0,72
Roseburia intestinalis DSM14610	NO	Firmicutes	Roseburia	1,09	LYBHI 4 RJ	NA	NA	NA	NA	NA	NA	0,57
Roseburia intestinalis DSM14610	NO	Firmicutes	Roseburia	0,45	M104	NA	NA	NA	NA	NA	NA	0,66
Roseburia inulinivorans DSM16841	NO	Firmicutes	Roseburia	0,46	LYBHI 4	106,610	28,980	3,200	15,590	5,990	2,900	0,37
Roseburia inulinivorans DSM16841	NO	Firmicutes	Roseburia	0,90	LYBHI 4 RJ	13,420	5,480	0,350	1,890	0,690	0,300	1,60
Roseburia inulinivorans DSM16841	NO	Firmicutes	Roseburia	0,63	LYBHI 4 RJ	2,260	0,870	0,000	0,160	0,000	0,000	0,74
Roseburia inulinivorans DSM16841	NO	Firmicutes	Roseburia	NA	M104	-4,610	0,000	0,000	4,180	0,000	0,000	0,97
Roseburia inulinivorans DSM16841	NO	Firmicutes	Roseburia	0,94	M412	-0,350	0,000	0,000	3,490	0,000	0,000	0,93
Ruminiclostridium leptum DSM753	NO	Firmicutes	Ruminiclostridium	0,22	LYHBHI 1	4,230	0,100	-0,170	-0,170	0,020	0,000	NA
Ruminiclostridium leptum DSM753	NO	Firmicutes	Ruminiclostridium	0,63	LYBHI 4 RJ	4,170	0,180	-0,020	-0,160	-0,080	0,000	1,15
Ruminiclostridium leptum DSM753	NO	Firmicutes	Ruminiclostridium	0,56	LYBHI 4 RJ	-2,120	-1,320	-0,090	-0,870	-0,190	-0,120	0,77
Ruminiclostridium leptum DSM753	NO	Firmicutes	Ruminiclostridium	0,56	LYBHI 4 RJ	6,300	0,480	0,010	-0,020	0,000	-0,120	0,94
Ruminiclostridium leptum DSM753	NO	Firmicutes	Ruminiclostridium	NA	LYBHI 4	3,920	0,200	-0,010	0,060	0,070	0,000	0,88
Ruminiclostridium leptum DSM753	NO	Firmicutes	Ruminiclostridium	0,56	LYBHI 4	4,530	0,100	-0,040	0,050	-0,470	0,000	1,26
Ruminiclostridium leptum DSM753	NO	Firmicutes	Ruminiclostridium	0,22	LYHBHI 1	6,420	0,100	0,030	0,070	0,020	0,000	0,91
Ruminiclostridium siraeum DSM3996	NO	Firmicutes	Ruminiclostridium	0,29	LYBHI 4 RJ	100,450	29,340	3,220	14,980	6,260	2,860	1,01
Ruminiclostridium siraeum DSM3996	NO	Firmicutes	Ruminiclostridium	0,70	LYBHI 4 RJ	NA	NA	NA	NA	NA	NA	0,99
Ruminiclostridium siraeum DSM3996	NO	Firmicutes	Ruminiclostridium	0,46	LYBHI 4 RJ	9,260	1,550	0,000	1,010	0,120	0,060	0,97
Ruminiclostridium siraeum DSM3996	NO	Firmicutes	Ruminiclostridium	0,66	M110 RJ	3,480	0,000	0,060	0,010	0,030	0,000	1,06
Ruminiclostridium siraeum DSM3996	NO	Firmicutes	Ruminiclostridium	0,85	M110 RJ	-3,480	-1,430	0,000	-0,840	-0,300	-0,120	1,19
Ruminiclostridium siraeum DSM3996	NO	Firmicutes	Ruminiclostridium	0,17	M104	15,700	1,530	0,000	1,020	0,460	0,070	1,00
Ruminiclostridium sporosphaeroides DSM1294	NO	Firmicutes	Ruminiclostridium	1,85	LYBHI 1	19,770	0,130	0,050	0,200	0,100	NA	1,05
Ruminiclostridium sporosphaeroides DSM1294	NO	Firmicutes	Ruminiclostridium	0,12	LYBHI 1	26,070	0,900	0,000	6,450	-0,070	0,000	0,47
Ruminiclostridium sporosphaeroides DSM1294	NO	Firmicutes	Ruminiclostridium	1,09	LYBHI 1	10,670	0,330	0,000	2,310	-0,060	0,000	0,56
Ruminiclostridium sporosphaeroides DSM1294	NO	Firmicutes	Ruminiclostridium	1,02	LYBHI 1	27,530	1,280	0,000	7,480	-0,070	0,000	0,61
Ruminiclostridium sporosphaeroides DSM1294	NO	Firmicutes	Ruminiclostridium	0,97	M78	16,170	11,665	-0,045	0,765	-0,093	-0,025	0,78
Ruminiclostridium sporosphaeroides DSM1294	NO	Firmicutes	Ruminiclostridium	1,85	M78	3,940	-0,405	-0,075	0,165	-0,053	-0,025	1,18
Ruminiclostridium sporosphaeroides DSM1294	NO	Firmicutes	Ruminiclostridium	0,46	M78	50,230	18,960	0,060	4,750	0,180	0,000	0,54
Ruminiclostridium sporosphaeroides DSM1294	NO	Firmicutes	Ruminiclostridium	1,48	M110	NA	NA	NA	NA	NA	NA	0,60
Ruminiclostridium sporosphaeroides DSM1294	NO	Firmicutes	Ruminiclostridium	1,06	M78	NA	NA	NA	NA	NA	NA	0,64
Ruminiclostridium sporosphaeroides DSM1294	NO	Firmicutes	Ruminiclostridium	1,35	LYBHI 4 RJ	NA	NA	NA	NA	NA	NA	0,72
Ruminococcus bromii ATCC27255	NO	Firmicutes	Ruminococcus	1,99	LYBHI 4	3,840	0,580	0,100	0,350	-0,240	0,110	1,01
Ruminococcus bromii ATCC27255	NO	Firmicutes	Ruminococcus	1,98	LYBHI 4	4,390	0,290	0,030	0,200	0,080	0,000	1,00
Ruminococcus bromii ATCC27255	NO	Firmicutes	Ruminococcus	1,89	LYBHI 4	4,190	0,260	0,050	0,200	0,100	0,000	0,77
Ruminococcus bromii ATCC27255	NO	Firmicutes	Ruminococcus	2,00	LYBHI 4 RJ	3,680	0,650	0,080	0,180	0,080	0,010	1,09
Ruminococcus bromii ATCC27255	NO	Firmicutes	Ruminococcus	1,92	LYBHI 4 RJ	-0,640	0,340	0,030	0,280	0,010	0,000	1,00
Ruminococcus bromii ATCC27255	NO	Firmicutes	Ruminococcus	1,96	LYBHI 4	1,950	0,140	0,020	0,190	0,100	0,000	0,90
Ruminococcus lactaris ATCC29176	NO	Firmicutes	Ruminococcus	1,40	LYBHI 1	9,900	0,280	0,050	0,220	0,080	0,000	0,81
Ruminococcus lactaris ATCC29176	NO	Firmicutes	Ruminococcus	1,48	LYBHI 4	9,410	0,420	0,050	0,290	-0,280	0,000	0,85
Ruminococcus lactaris ATCC29176	NO	Firmicutes	Ruminococcus	0,94	LYBHI 4	4,480	0,210	0,020	0,120	0,050	0,00	0,92
Ruminococcus lactaris ATCC29176	NO	Firmicutes	Ruminococcus	NA	LYBHI 4	3,710	0,100	0,000	0,060	0,010	0,00	0,93
Ruminococcus lactaris ATCC29176	NO	Firmicutes	Ruminococcus	0,97	LYBHI 4 RJ	7,740	0,510	0,060	0,280	0,160	0,01	0,88
Ruminococcus lactaris ATCC29176	NO	Firmicutes	Ruminococcus	0,49	LYBHI 4	4,830	0,400	0,060	0,290	0,170	0,00	0,84
Ruminococcus torques ATCC27756	NO	Firmicutes	Ruminococcus	0,32	M104	5,150	0,000	-1,360	-0,190	0,000	0,00	0,63
Sutterella parvirubra DSM19354	NO	Proteobacteria	Sutterellaceae	0,62	LYBHI 4	NA	NA	NA	NA	NA	NA	0,74
Sutterella parvirubra DSM19354	NO	Proteobacteria	Sutterellaceae	0,96	LYBHI 1	NA	NA	NA	NA	NA	NA	0,83
Sutterella wadsworthensis DSM14016	NO	Proteobacteria	Sutterellaceae	0,07	LYBHI 4 RJ	NA	NA	NA	NA	NA	NA	1,09
Sutterella wadsworthensis DSM14016	NO	Proteobacteria	Sutterellaceae	NA	M110 RJ	NA	NA	NA	NA	NA	NA	0,77
Sutterella wadsworthensis DSM14016	NO	Proteobacteria	Sutterellaceae	0,37	LYBHI 4 mucin	NA	NA	NA	NA	NA	NA	1,06
Tyzzerella nexilis DSM1787	NO	Firmicutes	Tyzzerella	1,34	M110	4,010	-0,285	-0,045	-0,415	0,308	-0,03	0,81
Tyzzerella nexilis DSM1787	NO	Firmicutes	Tyzzerella	0,65	M110	2,800	-0,355	-0,045	-0,275	0,738	-0,03	1,13
Tyzzerella nexilis DSM1787	NO	Firmicutes	Tyzzerella	0,37	M78	-5,330	-0,315	-0,075	-0,255	0,007	-0,03	1,05

Tyzzerella nexilis DSM1787	NO	Firmicutes	Tyzzerella	0,30	LYBHI 1	6,170	0,000	-0,150	-0,200	0,000	0,00	0,90
